# Conformational Quenching in an Engineered Lipocalin Protein Achieves High Affinity Binding to the Toxin Colchicine

**DOI:** 10.1002/anie.202515950

**Published:** 2025-10-27

**Authors:** Mark J. Bostock, Christopher Kolloff, Elena Jerschke, Sam Asami, Arne Skerra, Simon Olsson, Michael Sattler

**Affiliations:** ^1^ Bavarian NMR Center and Department of Bioscience TUM School of Natural Sciences Technical University of Munich 85748 Garching Germany; ^2^ Institute of Structural Biology Molecular Targets and Therapeutics Center Helmholtz Munich 85764 Neuherberg Germany; ^3^ Department for Computer Science and Engineering Chalmers University of Technology and University of Gothenburg Rännvägen 6 Gothenburg SE‐41296 Sweden; ^4^ Chair of Biological Chemistry, TUM School of Life Sciences Technical University of Munich 85354 Freising Germany

**Keywords:** Anticalin, Conformational selection, Molecular dynamics, NMR spectroscopy, Protein dynamics

## Abstract

The engineered lipocalin Colchicalin binds the clinically relevant plant toxin colchicine with picomolar affinity. X‐ray structures revealed major loop rearrangements at the open end of the β‐barrel upon ligand binding, suggesting a critical role for protein dynamics. Here, we integrated solution NMR relaxation experiments with molecular dynamics (MD) simulations and Markov modelling to examine conformational dynamics in the free and ligand‐bound Colchicalin on the picosecond‐to‐millisecond timescale. Fast backbone dynamics were comparable in the presence and absence of colchicine, indicating preserved secondary structure. However, large‐scale fluctuations in the structurally variable loops on the microsecond‐to‐millisecond timescale were observed in the apo form. We identified conformational exchange between three states, binding competent, partially closed and fully closed, characterised by loop L3 rearrangements. Colchicine binding quenches these motions, indicating a strong interplay between protein dynamics and ligand recognition. Our results support conformational selection over induced fit as the binding mechanism, highlighting the critical role of slow‐timescale dynamics to enable specific, high‐affinity ligand recognition and providing an important example for rational drug design.

## Graphical abstract

Specific high affinity recognition of colchicine by an engineered anticalin involves loop dynamics and conformational selection

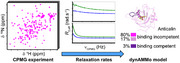

## Introduction

Lipocalins are small secreted proteins involved in the transport, scavenging and storage of biochemical molecules across diverse phyla including mammals, insects, plants and bacteria.^[^ ^1^ ^]^ In humans, more than a dozen distinct lipocalins have been identified.^[^ ^2^, ^3^ ^]^ The lipocalin fold comprises a central eight‐stranded antiparallel β‐barrel, with an amphipathic α‐helix leaning against one side, which is closed at one end by three tight loops. The other end of the β‐barrel is open to the solvent with four structurally variable loops, which form the binding pocket.^[^ ^4^, ^5^ ^]^ Directed protein evolution, involving techniques such as targeted mutagenesis, phage display and fluorescence‐activated cell sorting (FACS), has been used to develop anticalins, artificial lipocalins with specificity against a range of clinically relevant targets.^[^ ^6^, ^7^ ^]^ Several anticalins, especially those derived from the neutrophil gelatinase associated lipocalin (NGAL, also known as human lipocalin 2, Lcn2), a human plasma lipocalin, have reached clinical trials.^[^ ^8^, ^9^ ^]^

Recently an anticalin, Colchicalin D6.2, was developed based on the NGAL scaffold, which tightly binds the plant toxin colchicine (*K*_D_ = 120 pM).^[^ ^10^ ^]^ Colchicine is a plant secondary metabolite found in members of the lily family. It acts by binding to tubulin and inhibiting microtubule polymerisation. Applied at low doses, colchicine is anti‐inflammatory, for example through inhibition of neutrophil mobility, but at higher doses it can lead to fatal systemic toxicity due to its strong antimitotic activity.^[^ ^11^, ^12^, ^13^, ^14^ ^]^ In the search for an antidote that forms a stable complex and thus effectively blocks the toxic activity of colchicine, anticalins present an attractive therapeutic approach compared to antibodies, due to their small size, high expression yield in *Escherichia coli*, tight ligand binding via a deep pocket and low immunogenic potential.^[^ ^8^, ^9^ ^]^

The anticalin D6.2 (M69Q mutant) was initially crystallised in the colchicine‐bound state (PDB ID: 5NKN),^[^ ^10^ ^]^ which was followed by X‐ray structural analysis of the apo state (PDB ID: 6Z6Z).^[^ ^15^ ^]^ A comparison between the crystal structures of the Colchicalin‐colchicine complex and the natural lipocalin, Lcn2, bound to its ligand enterobactin (PDB ID: 3CMP),^[^ ^16^ ^]^ revealed that the overall lipocalin fold remained unperturbed (Figure S1). However, notable conformational differences were seen in the four structurally variable loop regions, which partially cover the ligand pocket in the colchicine complex. Compared with the wild‐type Lcn2, loop L2 is bent inward by up to 11.2 Å and contains a *cis*‐peptide bond at Pro72; loop L3 is bent inward by up to 6.3 Å while loop L4 is bent outward by up to 7.5 Å (Figure S1B).^[^ ^4^, ^10^, ^16^ ^]^ Only loop L1 remained largely unperturbed. The colchicine molecule is bound tightly packed in a deep pocket, surrounded by several aromatic side chains: Phe71, which forms a critical contact with the tropolone ring of colchicine, is conserved from Lcn2 and repositioned through the considerable conformational changes in loop L2, while other aromatic side‐chain interactions (Phe41, Phe68, Trp106, Phe134) were introduced during the directed evolution process.^[^ ^10^ ^]^

**Figure 1:**
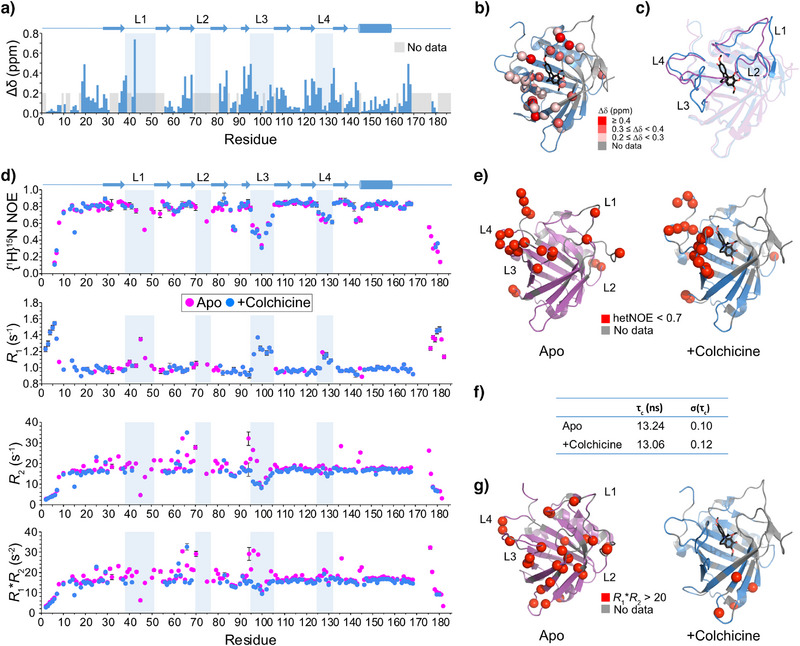
Comparison of the apo and colchicine‐bound states of Colchicalin by solution NMR. a) Chemical shift perturbations are plotted against residue number and b) highlighted as spheres on the X‐ray structure of the colchicine‐bound Colchicalin D6.2 (PDB ID: 5NKN). c) Crystal structures of the apo state (magenta, PDB ID: 6Z6Z) and the colchicine‐bound complex (blue, PDB ID:5NKN) are superimposed highlighting changes in the loop regions, in particular for L3. d) hetNOE, *R*_1_, *R*_2_ and *R*_1_*R_2_ values are plotted against residue number for the apo state (magenta) and the colchicine‐bound complex (blue) of Colchicalin. When errors are smaller than the radius of the data point, no error bars are shown. e) hetNOE values < 0.7, indicative of fast ps–ns motions, are shown as red spheres on the crystal structures of the apo state (magenta, PDB ID: 6Z6Z) and the colchicine‐bound complex (blue, PDB ID: 5NKN). f) Correlation times for the apo and colchicine‐bound states. g) *R*_1_*R_2_ values > 20 s^−2^ indicating slow (µs–ms) timescale motions are highlighted as red spheres on the crystal structures of the apo and colchicine‐bound states. Secondary structure elements are defined throughout according to the structural comparison in Achatz et al.^[^ ^4^ ^]^ Loop regions are defined as follows: L1, residues 38–51; L2, residues 70–76; L3, residues 95–105; L4, residues 125–132.

Comparison of the crystal structure of Colchicalin D6.2 in the apo state with the colchicine‐bound state showed that loops L1, L2, and L4 remain largely unchanged upon colchicine binding. In contrast, loop L3 shows a significant outward shift, with an RMSD for Cα positions of 6.3 Å, based on an alignment of the 58 Cα atoms of the β‐barrel,^[^ ^4^, ^10^, ^15^ ^]^ reaching the maximum Cα deviations at residues Ile97 (11.1 Å) and Lys98 (11.7 Å) (Figure 1c). Notably, both residues occupy the colchicine‐binding pocket in the apo state of Colchicalin and must be displaced for colchicine binding. Thus, the available crystal structures reveal substantial changes in the loop positions between the natural Lcn2 scaffold and the engineered Colchicalin in both its apo and colchicine‐bound states. This underscores the importance of the loop segments and their dynamics in ligand binding for this engineered lipocalin.

Small molecule binding to proteins is often associated with protein conformational changes, resulting from conformational dynamics in and around ligand‐binding pockets, which may promote access to the pocket and may be followed by further packing of the bound ligand. This often involves changes in side‐chain positions or bound water molecules and, more rarely, changes in the backbone conformation.^[^ ^17^ ^]^ Such phenomena are seen especially for hydrophobic ligands, which tend to be recognised in internal pockets rather than on the surface of a protein. Examples include fatty acid binding^[^ ^18^ ^]^ or drug binding to FK506‐binding proteins.^[^ ^19^, ^20^ ^]^ Understanding the role of protein dynamics in ligand binding is important to support rational drug design, with high affinity ligand binding typically requiring conformational changes on the target.^[^ ^21^, ^22^, ^23^ ^]^

In the present study, we investigated Colchicalin D6.2 in the presence and absence of its ligand colchicine using a range of NMR relaxation experiments. NMR relaxation data were combined with molecular dynamics (MD) simulations to gain atomistic insight into the mechanism of these motions using the dynamic Augmented Markov Model (dynAMMo) approach,^[^ ^24^, ^25^ ^]^ thus providing further understanding of the role of the loop regions and conformational dynamics of Colchicalin upon colchicine binding.

## Results and Discussion

### Probing Fast Timescale Dynamics in Colchicalin Using NMR Backbone Relaxation Analysis

Nearly‐complete backbone assignments of the apo state and colchicine‐bound state of Colchicalin were obtained using standard NMR techniques (see Supporting Information Methods) and deposited in the BMRB^[^ ^26^ ^]^ with accession codes 52911 and 52912, respectively. The ^1^H,^15^N HSQC NMR spectra showed good dispersion, although some signals were observed to be broadened in the colchicine‐bound state (Figure S2A).

Spectral comparison of the free and ligand‐bound states of the anticalin showed significant chemical shift perturbations (CSP) for several residues along the amino acid sequence, which cluster in the loop regions and at the termini of the β‐strands at the open end of the β‐barrel. Smaller changes were observed in the core of the protein and at the closed end of the β‐barrel, indicating the absence of major tertiary structure perturbations (Figure 1a,b). Secondary structure predictions using TALOS‐N^[^ ^27^ ^]^ were also comparable between the apo and colchicine‐bound states while the RCI‐S^2^ value^[^ ^28^ ^]^ demonstrated that colchicine binding does not result in a restriction of the fast timescale loop dynamics of the anticalin (Figure S3).

To analyse changes in the conformational dynamics upon colchicine binding, we first performed ^15^N backbone relaxation analysis using {^1^H}‐^15^N heteronuclear NOE (hetNOE), *R*_1_ and *R*_2_ measurements (Figure 1d). Calculation of *R*_2_/*R*_1_ was used to estimate the τ_c_, yielding values of 13.24 ± 0.10 ns (apo state) and 13.06 ± 0.12 ns (colchicine‐bound complex) (Figure 1f). This confirms that there are no large‐scale structural perturbations, which could influence the tumbling correlation time of the protein. {^1^H}‐^15^N hetNOE and ^15^N *R*_1_ measurements report on ps–ns timescale motions. Although chemical shifts of the central residues of the long L1 loop and the L2 loop could not be assigned, the pattern of hetNOE values indicated that the protein is well structured (hetNOE ∼0.8) while L3, L4 and two small regions around residues 88 and 143 as well as the N‐ and C‐termini showed faster motion (Figure 1d,e). A similar pattern was observed for the *R*_1_ data (Figure 1d). The hetNOE and *R*_1_ rates also showed almost no changes between the apo‐ and colchicine‐bound protein, indicating that colchicine binding does not impact the fast‐motion dynamics of Colchicalin.

^15^N *R*_2_ rates, which also report on fast‐timescale motions but are influenced by tumbling anisotropy and exchange motion, leading to elevated values, were likewise comparable between the apo‐ and colchicine‐bound states. However, larger deviations were observed with a cluster of elevated rates in the apo state at the N‐terminal ends of L2 and L3. To distinguish between elevated *R*_2_ values due to chemical exchange and motional anisotropy, *R*_1_*R_2_ was calculated, which removes the dependence on the latter.^[^ ^29^ ^]^ The same clusters of elevated values were observed as in the *R*_2_ data, indicating that these regions are likely to be affected by chemical exchange. Elevated *R*_1_*R_2_ values were more significant for the apo state and clustered at the N‐terminal ends of L2 and L3 (Figure 1d,g). Only a very small number of *R*_1_*R_2_ values > 20 s^−2^ were identified for the colchicine‐bound anticalin, located remote from the binding site (Figure 1g). These data suggest that although there are no restrictions on fast timescale motions due to colchicine binding, colchicine binding may reduce slower µs–ms timescale motions, suggesting a role for regions undergoing conformational exchange in colchicine binding.

### NMR Relaxation Dispersion Indicates Changes in µs–ms Dynamics Coupled to Ligand Binding

To probe the influence of colchicine binding on slow‐timescale motions in the anticalin, ^15^N‐CPMG data were collected. Data were fitted using the software Relax,^[^ ^30^, ^31^, ^32^ ^]^ residues were assigned to either the Carver–Richards model for slow timescale dynamics (µs–ms dynamics)^[^ ^33^ ^]^ or to ‘No exchange contribution', and values for *k*_ex_, p_A_ and Δω (ppm) were extracted (Figure S4).

**Figure 2:**
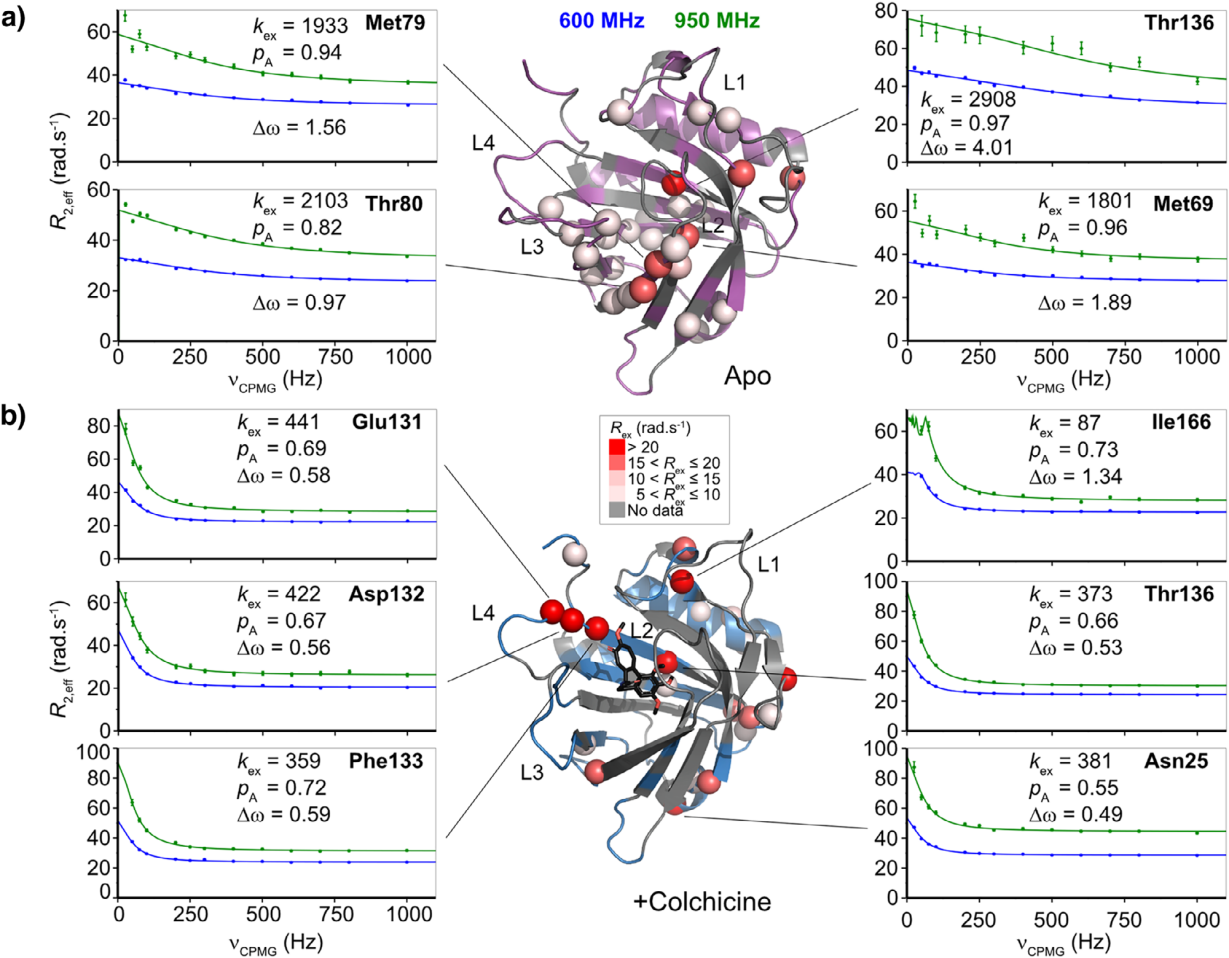
Microsecond‐to‐millisecond timescale backbone dynamics for Colchicalin in the apo and colchicine‐bound states. ^15^N CPMG relaxation dispersion profiles recorded at 600 MHz (blue) and 950 MHz (green) (^1^H frequency) are shown for the a) apo and b) colchicine‐bound states. *R*_ex_ contributions at 950 MHz > 5 rad·s^−1^ are plotted on the corresponding X‐ray structures as spheres and dispersion profiles for selected residues are shown. Fitted values for *k*_ex_ (rad·s^−1^), p_A_ (%) and Δ*ω* (ppm) are indicated on the graphs. Fitted values for all residues are shown in Figure S4.

Several residues showed exchange contributions, defined as *R*_ex_ >5 Hz, and these were plotted according to the magnitude of *R*_ex, 950 MHz_ onto the X‐ray structures of Colchicalin in the apo‐ and colchicine‐bound states (Figure 2a,b). The apo state had more small *R*_ex_ contributions around the β‐barrel, suggesting general slow‐timescale flexibility. In the apo state, loops L1, L3 and L4 all showed evidence of *R*_ex_ motions, along with the β‐strands adjacent to L2 (Figures 2a and S4). In the colchicine‐bound state, there were fewer residues with small *R*_ex_ contributions while there was an increase in residues showing larger *R*_ex_ contributions (>15 Hz). Compared to the apo state, clusters of *R*_ex_ contributions were seen at the base of the β‐barrel (Asn25, Phe27, His28, Gly29), in the loop connecting strands β6 and β7 (Gln117 and His118) and in the loop connecting β8 to the adjacent α‐helix (Arg140, Thr141, Glu143) as well as in the α‐helix itself (Leu148, Glu150, Ile153) (Figure 2b). The overlay of X‐ray structures (Figure 1c) and NMR‐derived secondary structure predictions (Figure S3) showed no significant structural changes in these regions.

Changes in relaxation dispersion contributions between the apo‐ and colchicine‐bound states may also reflect ring current shifts induced by the aromatic and tropolone rings in colchicine, thus affecting Δ*ω* as well as causing changes in protein dynamics. Notably, loop L3, which showed the largest structural rearrangement upon ligand binding according to the crystallographic analysis,^[^ ^10^, ^15^ ^]^ exhibited a cluster of residues with *R*_ex_ contributions in the apo state (Asn96, Lys98) along with residues in the adjacent β‐strands (Trp106, residues 108–110, Met120 and Val121). *R*_ex_ and *k*_ex_ contributions in L3 were reduced upon colchicine binding, indicating reduced dynamics at slow timescales, likely related to the displacement of L3 from the colchicine‐binding site. Lack of CPMG data in the colchicine‐bound state for L1 and L2 prevented comparison of these regions. However, a cluster of residues with high *R*_ex_ values was also observed in the colchicine‐bound state at the C‐terminal end of L4 and the N‐terminal end of strand β8 (Glu131, Asp132 and Phe133) (Figure 2b).

Overall, these data suggest a shift in *R*_ex_ contributions throughout the β‐barrel region of the anticalin with restrictions in some regions but increased µs–ms motions in others. Notably, the overall trend from the apo‐ to the colchicine‐bound state was a decrease in *k*_ex_, indicating reduced motional dynamics upon ligand binding (Figure S4).

**Figure 3:**
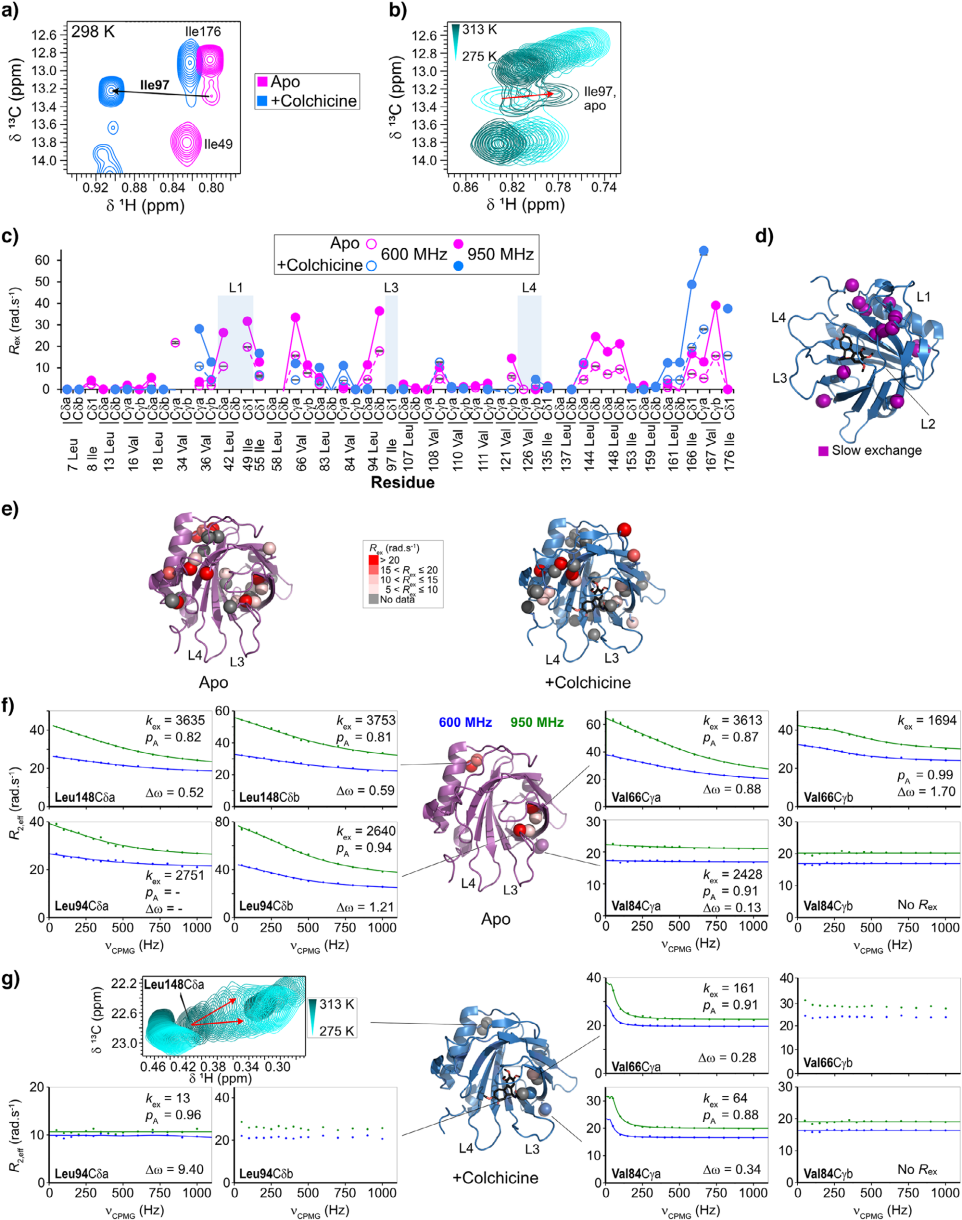
NMR analysis of microsecond‐to‐millisecond timescale sidechain methyl dynamics for ILV‐^1^H,^13^C‐methyl‐labelled Colchicalin in the apo and colchicine‐bond states. Substantial chemical shift perturbations are observed for Ile97 between the two states. a) Ile97 undergoes chemical exchange in the apo state, with b) a temperature series showing that the residue is in slow exchange at low temperature (275 K), shifting to a broad signal at higher temperatures (313 K), indicating a transition to the intermediate exchange regime (red arrow). c) *R*_ex_ contributions from ^13^C‐methyl CPMG relaxation dispersion experiments at 298 K are shown for the apo (pink) and colchicine‐bound (blue) states. d) Residues observed in slow exchange during a temperature series for the colchicine‐bound state are shown as purple spheres. e) *R*_ex_ contributions at 950 MHz > 5 rad·s^−1^ are plotted on the X‐ray structures as spheres for the apo state and the colchicine‐bound complex. ^13^C‐methyl CPMG relaxation dispersion profiles recorded at 600 MHz (blue) and 950 MHz (green) (^1^H frequency) at 298 K for selected residues are shown for f) the apo state and g) the colchicine‐bound state, with equivalent residues indicated as spheres on the corresponding X‐ray structures. Fitted values for *k*_ex_ (rad·s^−1^), p_A_ (%) and Δ*ω* (ppm) are indicated on the graphs. For Leu94 Cδb and Val66 Cγb (colchicine‐bound state, (g)) there are insufficient points to accurately fit the slope. In the colchicine‐bound state (g), Leu148 Cδa is observed in slow exchange across the temperature series, with peak splitting indicated by arrows.

To further investigate the influence of slow‐motion dynamics on ligand binding by Colchicalin, we exploited a selective isotope‐labelling approach using ^13^C methyl‐labelled Ile‐δ1, Leu‐δ and Val‐γ methyl groups, which act as reporters of side‐chain dynamics.^[^ ^34^ ^]^ 2D ^1^H,^13^C HMQC TROSY experiments showed excellent spectral quality, and resonances were assigned using a combination of TOCSY and NOESY experiments (see Supporting Information Methods) (Figure S2B). To aid assignment, a temperature series was acquired from 275 to 313 K. Interestingly, in the apo state, the signal for Ile97 (L3) is broadened at room temperature but becomes sharper at lower temperature, indicating intermediate exchange (Figure 3a,b). In contrast, in the presence of colchicine a sharp, intense signal was observed for Ile97, demonstrating that the residue is in fast exchange and indicating a clear impact of colchicine binding on its side‐chain dynamics (Figure 3a).

Compared to Ile97, other methyl reporters were observed as single sharp peaks in the apo state with one residue, Val121 Cγb (β7), shifting into intermediate exchange below 283 K. In contrast, in the colchicine‐bound state, several residues were observed in slow exchange (peak splitting) during the temperature series while others revealed some peak broadening, suggesting a transition to intermediate exchange. These residues were found at the open end of the β‐barrel in loop L1, the adjacent β‐strand (β1) and the C‐terminus, at the C‐terminal end of β5 adjacent to loop L3 (Leu94 Cδb) and in the neighbouring β‐strand (Val84 Cγa and Cγb, β4) as well as at the base of the α‐helix that is attached to the β‐barrel (Leu144 Cδa, Leu148 Cδa and Cδb) (Figure 3d).

To provide a more quantitative analysis of the side‐chain dynamics, ^13^C single quantum CPMG relaxation dispersion experiments were recorded at two field strengths (600 MHz and 950 MHz) to probe slow‐motion (µs–ms timescale) dynamics. *R*_ex_ contributions at 298 K and 950 MHz are displayed in Figure 3c,e, with fitted parameters shown in Figure S5. Strong *R*_ex_ contributions were observed across the methyl reporters in the apo state, which were suppressed upon binding of colchicine, except for a few isolated residues, also clearly seen for the fitted *k*_ex_ (Figure S5). The notable exceptions were methyl groups of residues located close to the C‐terminus (Leu161, Ile166, Val167) indicating increased slow‐motion dynamics of the C‐terminal region of Colchicalin in the complex with colchicine. These residues exhibited peak splitting at lower temperatures, confirming the slow‐exchange dynamics (Figure 3c,d). Leu148 Cδa and Cδb, at the base of the α‐helix, showed a clear relaxation dispersion contribution in the apo state (Figure 3f). Upon binding of colchicine this reporter shifted into slow exchange, indicated by peak splitting (Figure 3g). Leu94 lies at the base of loop L3, pointing towards Val66 and Leu83, thus acting as a reporter for the motion in loop L3. Clear relaxation dispersion contributions were observed for this residue in the apo state (Figure 3f). These motions were lost or shifted to slower exchange rates in the colchicine‐bound state (Figure 3g). Furthermore, Leu94 Cδb in the colchicine‐bound state showed peak splitting below 283 K, confirming the transition to slow‐exchange dynamics. These results are consistent with the peak shapes observed in the HMQC experiments for Ile97, located within L3 (Figure 3a,b). In contrast to the general trend, Val84 Cγa showed an increased *R*_ex_ contribution in the colchicine‐bound state (Figure 3c,g). Val84 Cγa faces towards the solvent, and the observed *R*_ex_ contribution may result from a closer interaction with loop L3.

### Markov Modelling Reveals Three‐State Exchange in the Conformational Ensemble of Apo Colchicalin

To relate the experimental solution NMR dynamics data with atomic‐level structural changes, we performed 13 µs of cumulative MD simulations initialised from the X‐ray structures for the binding‐incompetent apo state of Colchicalin (PDB ID: 6Z6Z), which exhibits a closed loop L3 conformation, and 5.6 µs for the binding‐competent state of the ligand complex (having an open L3 conformation), with colchicine removed (PDB ID: 5NKN) (see Supporting Information Methods for details). We ran multiple adaptive rounds of simulation to characterise each of the intra‐state dynamics. Using these MD simulations, we were able to corroborate the slow dynamics of the anticalin observed in the experimental data. However, we did not observe exchange between the binding‐competent and incompetent conformations, which suggests that the exchange between both conformations is slow, on the order of ∼3 rad·ms^−1^. Nevertheless, combining our simulation with the experimental ^15^N backbone CPMG data allowed us to estimate a single global Markov state model, thus providing insight into the thermodynamics and kinetics of the conformational changes in the apo state at atomic resolution.

**Figure 4:**
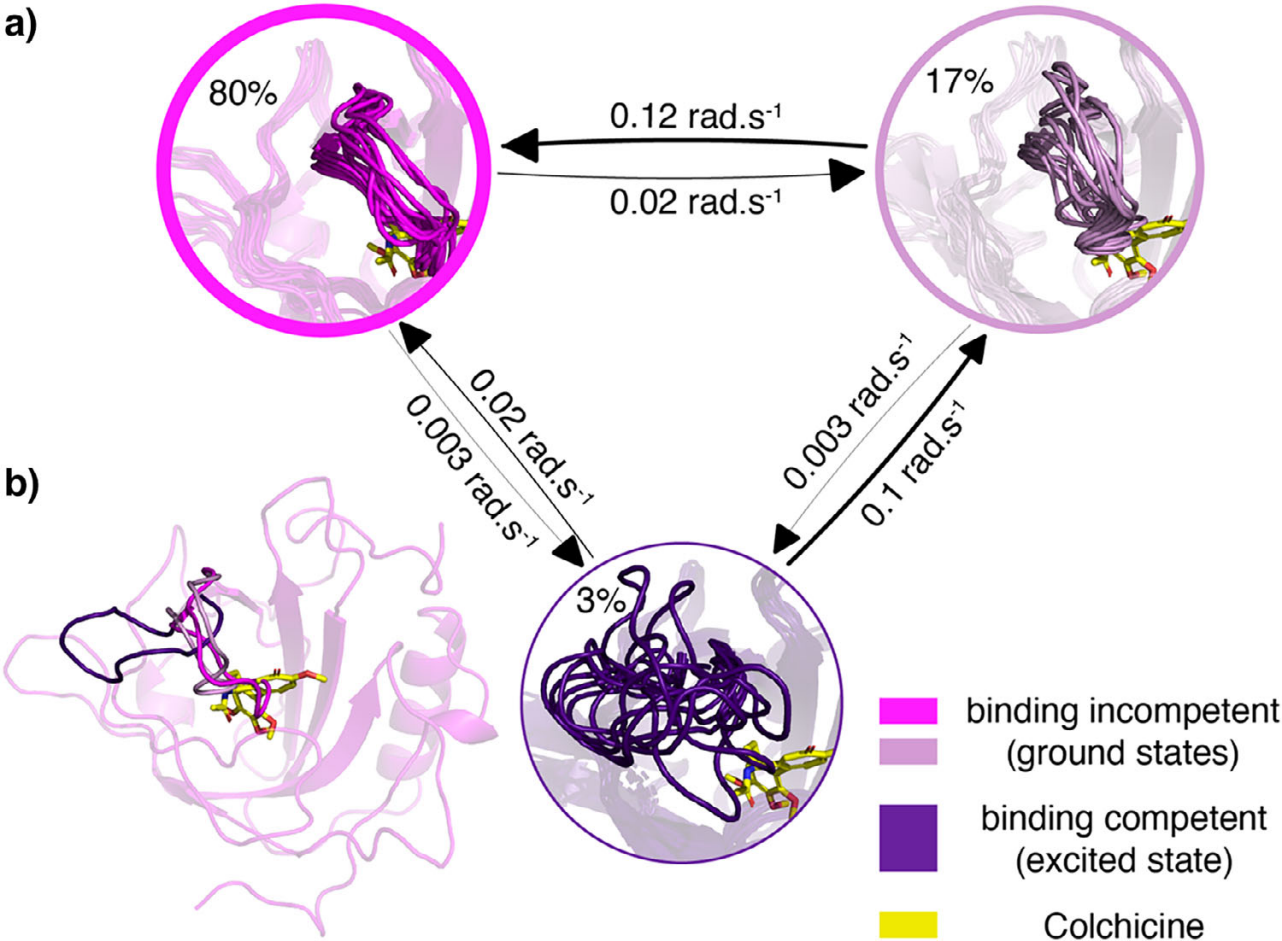
Overview of the dynamic Augmented Markov Model (dynAMMo) for the ligand‐free Colchicalin. a) Kinetic network derived from MD simulations and ^15^N CPMG relaxation dispersion data. The network is coarse‐grained into three states: one binding‐competent state (purple) and two binding‐incompetent states (dark and light pink). Relative populations of each state are indicated as percentages inside each circle. Bidirectional arrows connect the states, with exchange rates labeled in rad·s^−1^. Each state illustrates structural heterogeneity displayed as an ensemble of ten superimposed conformations. b) Full structure of the predominant binding‐incompetent state with loop L3 highlighted in all conformational states, with colours corresponding to those in (a). For illustration, the position of the ligand colchicine is shown in yellow overlaid on the apo state populations.

To characterise the exchange between the two conformations in the apo state of Colchicalin, we used the data to parametrise a dynamic Augmented Markov Model (dynAMMo) (Figure S6; see Supporting Information Methods for details).^[^ ^24^ ^]^ Utilising the ^15^N backbone relaxation dispersion data, we estimate the slow exchange between the two states to be in the fast millisecond regime (Figures 4a and S4 and S7). The exchange rates estimated by dynAMMo are consistent with the fits obtained using the standard Carver–Richards two‐state model (Figure S7). However, unlike *N*‐state model fits, typically used to analyse relaxation dispersion data, dynAMMo yields a Markov state model (MSM), which mechanistically describes the global chemical exchange process at atomic resolution and in turn allows for direct prediction of dynamic observables such as relaxation dispersion data.^[^ ^35^ ^]^ Consequently, unlike model fitting, dynAMMo does not rely on statistical testing and does not distinguish between local and global fitting of data but rather attempts to find the smallest perturbation of available simulation data to ensure agreement with the available experimental data. Therefore, the estimated dynAMMo summarises the available simulation and NMR data of the apo state of Colchicalin.

In simulations of the binding‐incompetent state, we sampled two predominant conformations: one completely occludes the binding pocket (Figure 4, dark pink, corresponding to PDB ID: 6Z6Z), whereas the other shows a slightly more extended loop L3 (Figure 4, light pink). Together, our dynAMMo model assigns 97% of the total population to the binding‐incompetent state. Although the transition between the fully closed and partially closed states was not reversibly sampled, the exchange was also estimated to be slow (Figure 4a) by our dynAMMo model. The binding‐competent state (Figure 4, purple, corresponds to PDB ID: 5NKN) was estimated to be approximately 3% populated and is characterised by the flexibility of loop L3, which opens the binding pocket allowing ligand access. In comparison to the flexible, highly dynamic L3 conformation in the low‐population state, the loop appears comparatively rigid in both binding‐incompetent states.

To compare the (fast) internal dynamics of the binding‐incompetent (fully and partially closed) and binding‐competent (open) states, we calculated backbone ^15^N *S^2^* order parameters.^[^ ^36^ ^]^ Comparing both sets of simulations, we observed similar fast dynamics across the protein with a tendency for the binding‐competent state to be slightly less structured and thus more flexible (Figure S8). However, the binding‐competent state showed significant flexibility in L3 compared to the two binding‐incompetent states, where loop L3 predominantly blocks the ligand‐binding pocket of the anticalin (Figure 4). This observation supports the hypothesis that L3 helps to stabilise the protein structure in the apo state, compensating for the missing colchicine that is otherwise deeply buried in the ligand pocket. This is corroborated by the NMR data where Ile97 Cδ1 is observed in intermediate exchange on the NMR chemical shift timescale in the apo state, consistent with slow exchange between multiple conformations, whereas in the ligand‐bound form, Ile97 Cδ1 is observed in fast exchange, consistent with displacement from the ligand pocket (Figure 3a,b).

### The Colchicalin L3 Loop Dynamically Samples Open and Closed Conformations

**Figure 5:**
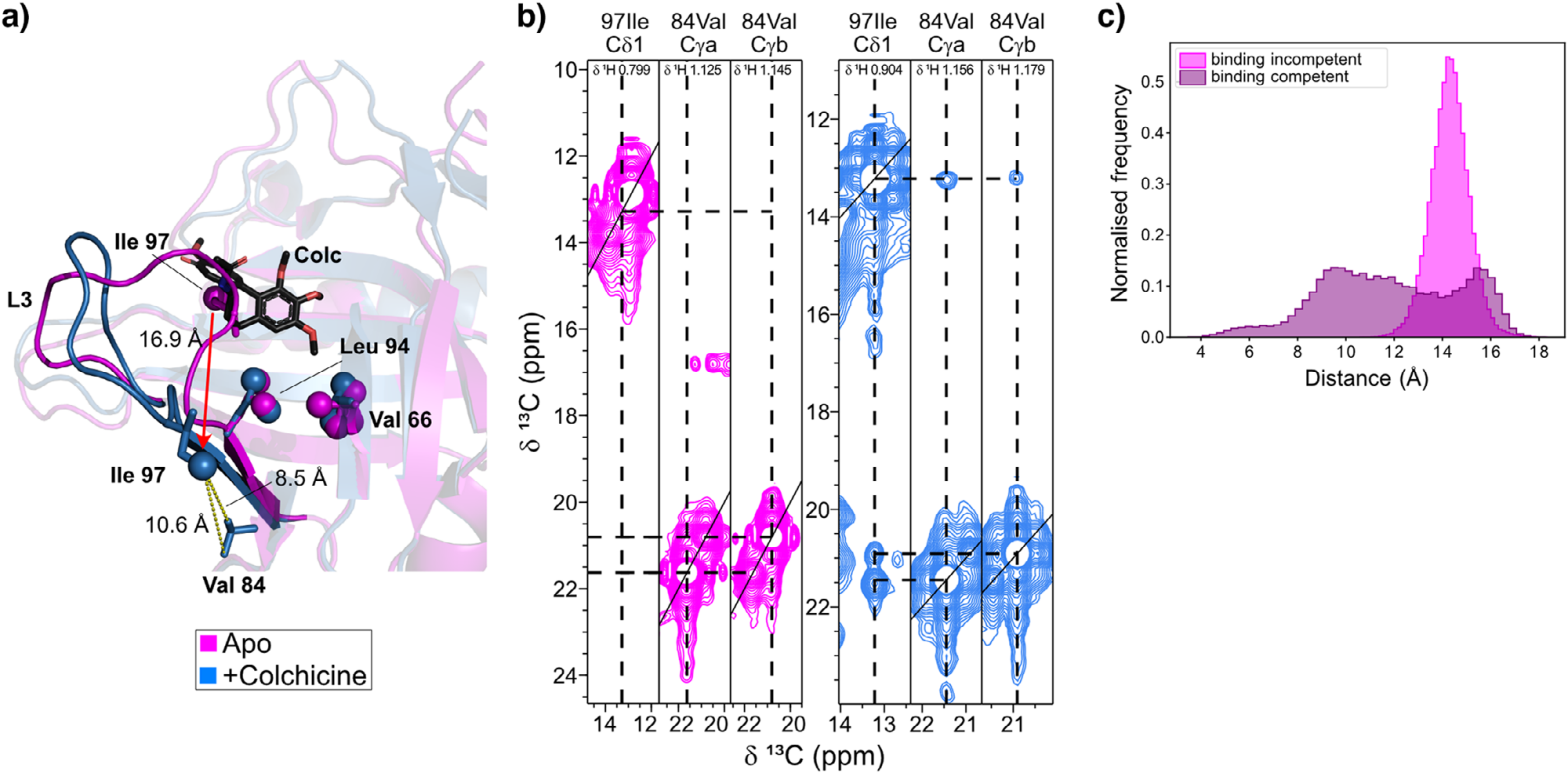
Conformational change in the L3 loop of Colchicalin upon binding colchicine. a) Key reporter residues observed in this study are shown on the superimposed X‐ray structures of the apo state (magenta) and the colchicine‐bound complex (blue) as sticks (PDB IDs 6Z6Z and 5NKN, respectively), with methyl groups illustrated as spheres. Ile97 Cδ1 undergoes a 16.9 Å shift between the apo and colchicine‐bound states. In the crystal structure of the colchicine‐bound complex, distances between Ile97 Cδ1 and Val84 Cγ1 and Cγ2 are 8.5 and 10.6 Å, respectively. b) CCH‐NOESY strips in the apo (magenta) and colchicine‐bound states (blue) for Ile97 Cδ1 and Val84 Cγa and Cγb, showing the presence of an NOE between these residues only in the colchicine‐bound state. c) Distance histograms between Ile97 Cδ1 and the geometric centre between Val84 Cγ1 and Cγ2 in the MD trajectories. The binding‐competent trajectories (purple) of the open states sample a wide range of distances, notably with a small population between 4 and 6 Å and an r*^−6^*‐averaged distance of 8.6 Å, consistent with the observed NOE. The binding‐incompetent trajectories (magenta) of the closed states sample a unimodal distribution of distances with an *r^−6^*‐averaged distance of 14 Å.

Our NMR relaxation data showed notable µs–ms dynamics in the loop regions, in particular exchange broadening for Ile97 in L3. Crystallographic analyses showed that L3 is displaced from the ligand pocket in the colchicine‐bound complex with a Cα RMSD of 6.3 Å, and elevated crystallographic B factors for L3 were observed in the colchicine‐bound Colchicalin,^[^ ^10^ ^]^ consistent with displacement of this loop upon colchicine binding: in the apo state the loop is observed in a closed conformation with Ile97 occupying the colchicine binding site, whereas in the presence of colchicine the loop appears to be pushed away from the pocket with Ile97 pointing into the solvent, indicated by a shift of 11.1 Å for Ile 97 Cα (16.9 Å for Cδ1) and 11.7 Å for Lys 98 Cα (Figure 5a).

Further data from CCH‐NOESY experiments were acquired for the apo‐ and colchicine‐bound states of Colchicalin. Notably, in the apo state no NOEs were observed to/from Ile97 Cδ1, while NOEs were detected from Ile97 Cδ1 to Val84 Cγa and Val84 Cγb, in the colchicine‐bound state (Figure 5b). NOEs observed between Ile97 Cδ1 and Val84 Cγa and Cγb in the complex with colchicine but not the apo state (Figure 5b) support the significant structural shift of L3, with distances between the methyl carbons of 8.5 and 10.6 Å measured for the colchicine‐bound X‐ray structure (Figure 5a). Observation of NOEs over longer distances is expected for methyl–methyl NOEs, typically in the range 4–10 Å.^[^ ^37^ ^]^ In addition, MD trajectories starting from the colchicine‐bound crystal structure showed a small population where the interatomic distances between these two residues are 4–6 Å in the binding‐competent state, comprising the open loop conformation, with an overall *r^−6^*‐averaged distance of 8.6 Å. In contrast the *r^−6^*‐averaged distances between Ile97 and Val84 are around 14 Å in the binding‐incompetent states, comprising the closed loop conformation (Figure 5c). This population of shorter distances in the binding‐competent state, which is representative of the colchicine‐bound Colchicalin, is likely responsible for the observed NOE, whereas the longer distances observed in the binding‐incompetent state (*r^−6^*‐averaged distance of 14 Å) are consistent with the lack of an NOE in the NOESY data of the anticalin in the absence of colchicine (Figure 5b).

Our NMR experiments also showed clear relaxation dispersion effects and large *R*_ex_ values for Leu94 Cδa and Cδb (C‐terminal end of β5 adjacent to L3) in the apo state, which were lost upon colchicine binding. In the colchicine‐bound form, peak splitting was observed for Leu94 at lower temperatures, indicating slow exchange on the NMR timescale. Moreover, line‐broadening (at room temperature) seen for the Ile97 Cδ1 signal in the ^13^C‐HMQC spectrum of the apo state is consistent with intermediate exchange, indicating that Ile97 exists in multiple conformations. Binding of colchicine reduces these motions by displacing Ile97 from the ligand pocket causing a shift into fast exchange and affecting the dynamics of residues adjacent to L3. Val66 and Leu94 both show motions shifted to slower timescales upon colchicine binding. While in the X‐ray structures the side chain of Leu94 did not significantly alter its conformation due to colchicine binding, this residue acts as a reporter of the changes occurring in loop L3. Similarly, Val66, in the adjacent β‐strand (β3), faces Leu94 and may also be impacted by the shifting motional regime for L3 (Figure 5a). Since the L3 loop conformation sterically prevents entry of colchicine into the ligand pocket of the binding‐incompetent conformations of the Colchicalin apo state but the apo state also samples a binding‐competent conformation, our data indicate that conformational selection is important for ligand binding, as previously postulated.^[^ ^15^ ^]^

## Conclusion

Our experimental NMR data and MD analysis reveal that Colchicalin is indeed a highly dynamic protein. Although no significant differences in ps–ns timescale motions were observed between the apo and colchicine‐bound states, substantial changes take place on the µs–ms timescale. The apo state shows significant µs–ms timescale motions, which are reduced or shifted to slow exchange upon binding of colchicine, indicating that complex formation causes tightening of the anticalin structure. This suggests a mechanism of entropy/enthalpy compensation, where the entropy loss associated with colchicine binding within the loop region (providing favourable enthalpy) is compensated by increased flexibility of residues at the base of the β‐barrel. In the apo state, the L3 loop blocks the ligand pocket, potentially stabilising the protein in the absence of its ligand. However, the L3 loop samples multiple conformations in solution, which are not seen in the crystal structures. Notably, our dynAMMo analysis of the apo state identified two binding‐incompetent populations, which fully and partially obstruct colchicine binding, as well as a low population state which is compatible with ligand binding. Exchange between multiple conformations of L3 exposes the ligand pocket to enable colchicine binding, indicating conformational selection as a likely mechanism of complex formation with Colchicalin.

Dynamic changes were also observed to be important for another lipocalin‐derived binding protein, FluA, with a reduction in slow motional modes observed in NMR relaxation experiments when in complex with fluorescein compared to the apo state, indicating rigidification of the β‐barrel upon ligand binding.^[^ ^38^ ^]^ Furthermore, the exchange of a dynamic loop between binding‐incompetent and binding‐competent states, which allows access of a ligand to the ligand‐binding cavity, is reminiscent of the binding of selective inhibitors to the FK506‐binding protein (FKBP51). In corresponding crystal structures, a Phe side chain partially occupies the ligand pocket in a binding‐incompetent state. However, NMR relaxation data showed that µs–ms timescale dynamics allow sampling of a transient binding‐competent state with the Phe side chain adopting an outward conformation, which enables the ligand to enter the pocket.^[^ ^20^ ^]^

In general, while crystallographic structures of free and ligand–bound proteins show the corresponding low energy conformations of these representative states, conformational dynamics play a crucial role in the binding mechanism, enabling specific recognition and formation of a high‐affinity complex. Information about conformational dynamics especially at µs–ms timescales from combined experimental and computational data is not only required to understand binding mechanisms, but may also aid the design of selective protein‐ligand interactions relevant to drug discovery.^[^ ^21^, ^22^, ^23^ ^]^

## Author Contributions

E.J. carried out molecular biology, protein expression and purification. M.J.B. carried out NMR measurements and analysis, with support from S.A. for experiment setup. C.K. and S.O. carried out MD simulations and dynAMMo analysis. M.J.B., C.K., S.O and E.J. prepared the first manuscript draft. M.J.B. and C.K. produced the figures. The manuscript was revised by all authors. M.S., S.O. and A.S. supervised the project.

## Funding

Deutsche Forschungsgemeinschaft, SFB1035 (project number 201302640) (to M.S.). The Wallenberg AI, Autonomous Systems and Software Program (WASP) funded by the Knut and Alice Wallenberg Foundation (to S.O and C.K). National Academic Infrastructure for Supercomputing in Sweden (NAISS) at Alvis (project: SNIC 2021/7–2 and SNIC 2022/22–57, to S.O.), partially funded by the Swedish Research Council through grant agreement no. 2022–06725 provided resources to enable simulations.

## Conflict of Interests

The authors declare no conflict of interest. M.J.B. is a current employee of AstraZeneca and has stock ownership and/or stock options or interests in the company.

## Supporting information



Supporting Information

## Data Availability

All data needed to evaluate the conclusions in the paper are present in the paper and/or the Supporting Information. NMR assignments have been deposited in the BMRB under accession numbers 52911 for the anticalin apo form and 52912 for the colchicine‐bound form.
