# Supplementary material for: Conformational Quenching in an Engineered Lipocalin Protein Achieves High Affinity Binding to the Toxin Colchicine
Source: Angew Chem Int Ed Engl. 2025 Oct 27;64(50):e202515950. doi: 10.1002/anie.202515950 (PMC12684335; doi:10.1002/anie.202515950)
Supplement: Supplementary file 1 — Supporting Information [file ANIE-64-e202515950-s001.pdf]

Supporting Information

Conformational Quenching in an Engineered Lipocalin Protein Achieves High Affinity Binding to the Toxin Colchicine

Mark J. Bostock[a,b*], Christopher Kolloff[c*], Elena Jerschke[d*], Sam Asami[a], Arne Skerra[d], Simon Olsson[c], Michael Sattler[a,b]

[a] Bavarian NMR Center and Department of Bioscience, TUM School of Natural Sciences, Technical University of Munich, 85748 Garching, Germany

[b] Institute of Structural Biology, Molecular Targets and Therapeutics Center, Helmholtz Munich, 85764 Neuherberg, Germany

[c] Department for Computer Science and Engineering, Chalmers University of Technology and University of Gothenburg, Rännvägen 6, Gothenburg SE-41296, Sweden

[d] Chair of Biological Chemistry, TUM School of Life Sciences, Technical University of Munich, 85354 Freising, Germany

*These authors contributed equally to this work.

Corresponding authors: Michael Sattler, michael.sattler@helmholtz-munich.de; Simon Olsson, simonols@chalmers.se; Arne Skerra, skerra@tum.de

Methods

Protein expression and purification

Protein expression and purification was carried out according to previously published procedures adapted for NMR using stable isotope labelling.[1,2] In brief, the coding region of Colchicalin D6.2 with a C-terminal His_6-tag was cloned on the pASK75-T7RBS2 vector, a derivative of pASK75.[3] Uniformly ^{15}N,^{13}C-labelled protein was prepared by transforming *E. coli* Origami B cells, which were grown in 1 mL LB medium supplemented with ampicillin (100 µg/mL), followed by overnight growth in 50 mL M9 minimal medium containing 0.5 g/L $^{15}NH_4Cl$ and 2 g/L ^{13}C-glucose, supplemented with ampicillin (100 µg/mL). This culture was used to inoculate 2 L of the same M9 medium at 37 °C. Cells were grown until $OD_{550} \approx 0.3$, at which point the temperature was reduced to 30 °C until $OD_{550} \approx 0.5$ was reached. Expression was induced with anhydrotetracycline (200 µg/mL) overnight at 30 °C. Cells were harvested by centrifugation, resuspended in buffer A (40 mM Na-P_i, 500 mM NaCl, pH 7.5) and lysed using a French pressure cell. The lysate was clarified by centrifugation (1 h, 38724 xg) and applied to a 5 mL Ni(II)-charged HisTrap HP column (GE Healthcare, Freiburg, Germany) equilibrated with buffer A. After washing, the bound protein was eluted with a linear concentration gradient from 4–40% buffer B (40 mM Na-P_i, 500 mM NaCl, 500 mM imidazole-HCl, pH 7.5). Fractions containing Colchicalin D6.2-His_6 were pooled and Na-EDTA pH 8.0 was added to a final concentration of 10 mM. After dialysis twice against buffer C (20 mM Tris-HCl, pH 8.5) at 4 °C the protein solution was centrifuged (20 min, 4324 xg) and purified by anion exchange chromatography (AEX) on a 6 mL Resource Q column (GE Healthcare) with a 0–50% concentration gradient from buffer C to buffer D (20 mM Tris-HCl, 1 M NaCl, pH 8.5). Relevant fractions were pooled, concentrated and purified using a Superdex 75 HiLoad 26/60 column (GE Healthcare) equilibrated with elution buffer (10 mM Na-P_i, 150 mM NaCl, 0.2 mM EDTA, pH 6.4). Fractions containing Colchicalin D6.2-His_6 were pooled and concentrated to 1 mM, yielding 17.5 mg labelled protein per 1 L bacterial culture. Protein purity was assessed by 15% (w/v) SDS-PAGE with Coomassie brilliant blue staining. The protein composition was verified using electrospray ionisation mass spectrometry (measured: 22672 Da; calculated: 22681 Da) in the positive ion mode on a maXis ESI-QTOF instrument (Bruker Daltonics, Bremen, Germany).

Preparation of the isoleucine/leucine/valine (ILV)-labelled Colchicalin D6.2 was carried out on a deuterated background. Bacteria were transformed as above and stepwise adapted to 50 mL deuterated M9 minimal medium supplemented with 2 g/L ^{12}C-glucose-D_7 at 37 °C in three steps (0%, 68%, 99.8% D_2O). 1 L M9 minimal medium, prepared using 99.8% D_2O, 0.5 g/L $^{15}NH_4Cl$ and 2 g/L ^{12}C glucose-D_7, was inoculated and grown at 37 °C. At $OD_{550} \approx 0.3$ the temperature was lowered to 32 °C until $OD_{550} \approx 0.4$ was reached, then 50 mg/L 2-keto-3,3-d_2-$^{13}C4$-butyrate and 100 mg/L 2-keto-3-(methyl-d_3)-butyric acid-4-^{13}C,3-d (NMR-Bio, Grenoble, France) were added to the medium. Expression was induced at $OD_{550} \approx 0.5$ with 200 µg/mL anhydrotetracycline and continued at 32 °C overnight. The ILV-labelled protein was purified as above, with the final size exclusion buffer prepared in D_2O, yielding 38 mg per 1 L bacterial culture. The protein composition was verified using SDS-PAGE and ESI-MS as above (measured: 22814 Da;

calculated: 23135 Da; for comparison, the calculated mass of the unlabelled protein, including one disulphide bond, is 21465 Da).

NMR spectroscopy

NMR experiments were recorded on Bruker Avance III 600 and 950 MHz (^{1}H frequencies, 600 & 950 MHz respectively) spectrometers (Bruker BioSpin, Ettlingen, Germany), equipped with cryogenic TCI or QCI triple resonance probes. All samples were prepared in phosphate buffer (10 mM Na-P$_i$, 150 mM NaCl, 0.2 mM EDTA, pH 6.4) at a protein concentration of ~1 mM. For assignment, [^{1}H,^{15}N] HSQC and three-dimensional (3D) heteronuclear HNCA, HN(CO)CA, HNCACB, CBCA(CO)NH, HNCO, HN(CA)CO and ^{15}N-edited ^{1}H nuclear Overhauser effect spectroscopy (NOESY) spectra (120 ms mixing time) were acquired on uniformly ^{15}N,^{13}C-labelled samples at 298 K.[4] For investigation of the anticalin-ligand complex a small excess of colchicine was added. Backbone assignment experiments (excluding the 3D ^{15}N-edited NOESY) were acquired with non-uniform sampling (NUS) using the default sample scheduler in Topspin software version 3.5 (Bruker Biospin, Germany) and reconstructed using the CS-IHT algorithm from the Cambridge CS software package.[5] Non-NUS processing was carried out using the *AZARA* software package (v.2.7, ©1993-2025; Wayne Boucher and Department of Biochemistry, University of Cambridge). All spectra were analysed using CcpNmr Analysis.[6] ^{15}N chemical shift perturbations (Δδ [ppm]) were calculated according to the formula:

$$\Delta\delta = \sqrt{(\Delta\delta_{HN})^2 + (0.15\ * \Delta\delta_{15N})^2}$$

^{15}N-backbone relaxation experiments were recorded at 600 MHz ^{1}H frequency and 298 K. {^{1}H}-^{15}N heteronuclear NOE (hetNOE) experiments were performed using a sequence with interleaved saturated and unsaturated planes with a 3 s saturation period.[7] NOE values were calculated as the ratio of intensities between the saturated and unsaturated planes. Errors were calculated from the standard deviation of the noise. ^{1}H,^{15}N HSQC-based ^{15}N R_1 and R_2 relaxation was measured using experiments based on sequences from Farrow *et al.*[7] with water-control during the relaxation period in the R_1 sequence using a cosine-modulated IBURP-2 pulse[8] and modifications in the R_2 sequences according to Lakomek *et al.,*[9]. Nine time points were used for the R_1 experiment (80*, 160, 240, 320*, 400, 640, 800, 1200 ms, with * recorded in triplicate) and seven time points for the R_2 experiment (14.4, 28.8*, 43.2, 57.6, 72.0*, 86.4, 100.8 ms, with * recorded in triplicate). Backbone μs–ms dynamics were studied using ^{15}N relaxation dispersion experiments using a SQ CPMG sequence.[10] Spectra were acquired at 600 MHz and 950 MHz ^{1}H frequency at 298 K. The CPMG field, ν_{CPMG}, was varied from 25 to 1000 Hz, with a T_{rel} of 40 ms and a 2 s recycle delay. Spectra were acquired in an interleaved format and error analysis used the RMSD of the noise for each plane. ^{15}N 180° pulse strengths were 3.1 kHz.

Methyl resonances of the ILV-labelled Colchicalin were assigned using a combination of 3D (H)CC(CO)NH-TOCSY, H(CC)(CO)NH-TOCSY (12 ms mixing time) and CCH-TOCSY (13.9 ms mixing time) experiments.[4] Assignments were further validated using a CCH NOESY

experiment (148 ms mixing time)[11] and transferred between 275 K and 313 K using a temperature series of ten $^{1}H,^{13}C$ HMQC experiments recorded at 275, 278, 283, 288, 293, 298, 303, 308, 310 and 313 K. Slow-timescale µs-ms dynamics were analysed using a single-quantum methyl ^{13}C CPMG sequence, acquired at 600 MHz and 950 MHz (^{1}H frequency) at 298 K, based on the sequence by Lundström *et al.,*[12]. ν_{CPMG} was varied from 50 to 1,000 Hz, using a T_{rel} of 40 ms and a 1 s recycle delay. Spectra were acquired interleaved with error analysis based on duplicate spectra acquired for two ν_{CPMG} values. The rf pulse strength for the ^{13}C 180° pulses was 2.8 kHz.

Relaxation dispersion analysis

CPMG data were fit using the programme Relax.[13–15] For ^{15}N CPMG experiments, data were fit to two different motional models (no exchange, CR72 and CR72 full[16]). For ^{13}C CPMG experiments, data were fit to additional motional models (no exchange, CR72 and CR72 full,[16] LM63,[17] IT99,[18] and NS CPMG 2-site expanded[10]). Initial values were determined using a grid search and the best fit assigned according to a χ^2 calculation and Akaike's Information Criteria (AIC) model selection.[19] Error analysis was carried out using Monte-Carlo simulations ($n = 500$). Fits were inspected visually. For the ^{13}C data, fits were excluded if peak splitting was observed in the HMQC experiment at the equivalent temperature. For the ^{15}N data, points with $\Delta\omega > 5$ ppm or RMSE > 2.5 were excluded from the analysis due to poor quality fits. R_{ex} was calculated using the fitted curves as $R_{2,eff}(1000\ Hz) - R_{2,eff}(50Hz)$.

MD simulations

Molecular dynamics (MD) simulations were set up using the previously published Colchicalin ligand- (colchicine) bound state (PDB ID: 5NKN, Δ4-D6.2(M69Q)[1] and the crystal structure of apo-Colchicalin (PDB ID: 6Z6Z).[20] The experimental structures were prepared for simulation using PDBFixer, stripping crystallographic water molecules and colchicine, adding missing atoms at idealized positions, solvating the proteins in 100 mM NaCl buffer at pH 7 with TIP3P[21] water molecules (between 8286 – 9392 molecules, depending on the setup) using cubic box sizes ranging from 67.8 nm^3 to 69 nm^3. The protein was modelled with the AMBER14SB forcefield, non-bonded interactions were treated with particle mesh Ewald[22,23] beyond a 1 nm cutoff, and hydrogen mass repartitioning was used to enable stable long-time step production simulation. Following preparation, the systems were energy minimized using OpenMM,[24] and pre-equilibrated for 5 picoseconds, with a timestep of 0.5 femtoseconds at 300 K. The final coordinates were used to seed multiple molecular dynamics simulations run in parallel using OpenMM.[24] A total of 24 simulations was carried out, with a cumulative simulation time of 16.4 microseconds. The simulations were run in six successive batches where each batch was seeded with an initial configuration sampled randomly from the previous batch. Each production simulation was equilibrated for 40 picoseconds with a timestep of 2 femtoseconds. Production simulations ranged from 474 nanoseconds to 1.04 microseconds in length and were carried out with 4 femtosecond time steps. The trajectory configurations were saved every 200 picoseconds. All simulations were conducted in the NVT ensemble using Langevin dynamics.

Markov modelling

The simulation data were pooled together to build two Markov state models (MSMs)[25–27] separately for the two sets of simulations. Backbone and sidechain dihedrals together with inverse distances between residues Ile97, Val66, and Val84 were used as features for dimensionality reduction using time-lagged Independent Component Analysis (tICA).[28] The four slowest independent components (ICs) were used for discretising the state space into 100 clusters each using KMeans. The discrete trajectories were then used to build Markov models with a lag time of 20 ns, which were subsequently checked for convergence using implied timescale plots and verified using Chapman-Kolmogorov tests (Figure S6). All analyses were conducted using the deeptime library.[29]

The two (disconnected) Markov models were then used to initialise a dynamic Augmented Markov Model (dynAMMo).[30] dynAMMo allows the integration of information about intra-state dynamics from MD simulations with dynamic experimental observables sensitive to slow exchange between states, such as CPMG data, to build a Markov model. Importantly, model building with dynAMMo is possible even in our case where the very slow transition between the binding-competent and binding-incompetent states of the Colchicalin could not be observed during the simulations. The initial populations were scaled to 0.9 and 0.1, for the apo and holo MSMs, respectively. All other parameters for estimating the dynAMMo are listed in Table S1.

A

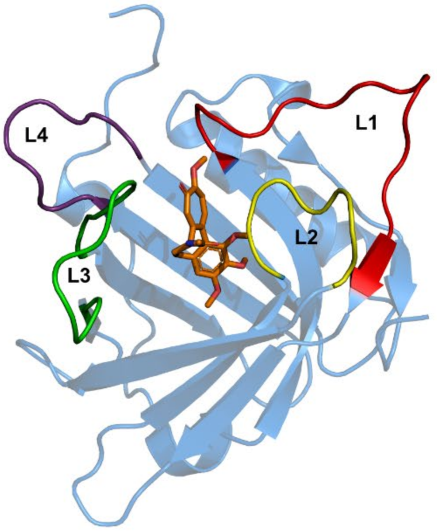

Colchicalin D6.2 + colchicine (PDB: 5NKN)

B

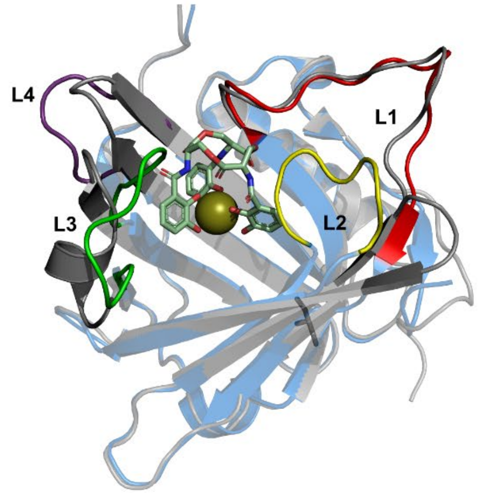

Lipocalin 2 + enterobactin (PDB: 3CMP)
Colchicalin D6.2 + colchicine (PDB: 5NKN)

Figure S1. Comparison of X-ray crystal structures for colchicine-bound Colchicalin D6.2 and enterobactin-bound lipocalin 2 (K125A mutant). (A) Colchicine-bound Colchicalin D6.2 (PDB ID: 5NKN) is shown in blue, with colchicine coloured orange. The four loop regions are highlighted, defined according to the structural comparison in Achatz *et al*.[31] as follows: L1, residues 38–51; L2, residues 70–76; L3, residues 95–105; L4, residues 125–132. (B) Overlay of colchicine-bound Colchicalin (blue, PDB ID: 5NKN) and enterobactin-bound lipocalin 2 (grey, PDB ID: 3CMP) with the anticalin loop regions highlighted.

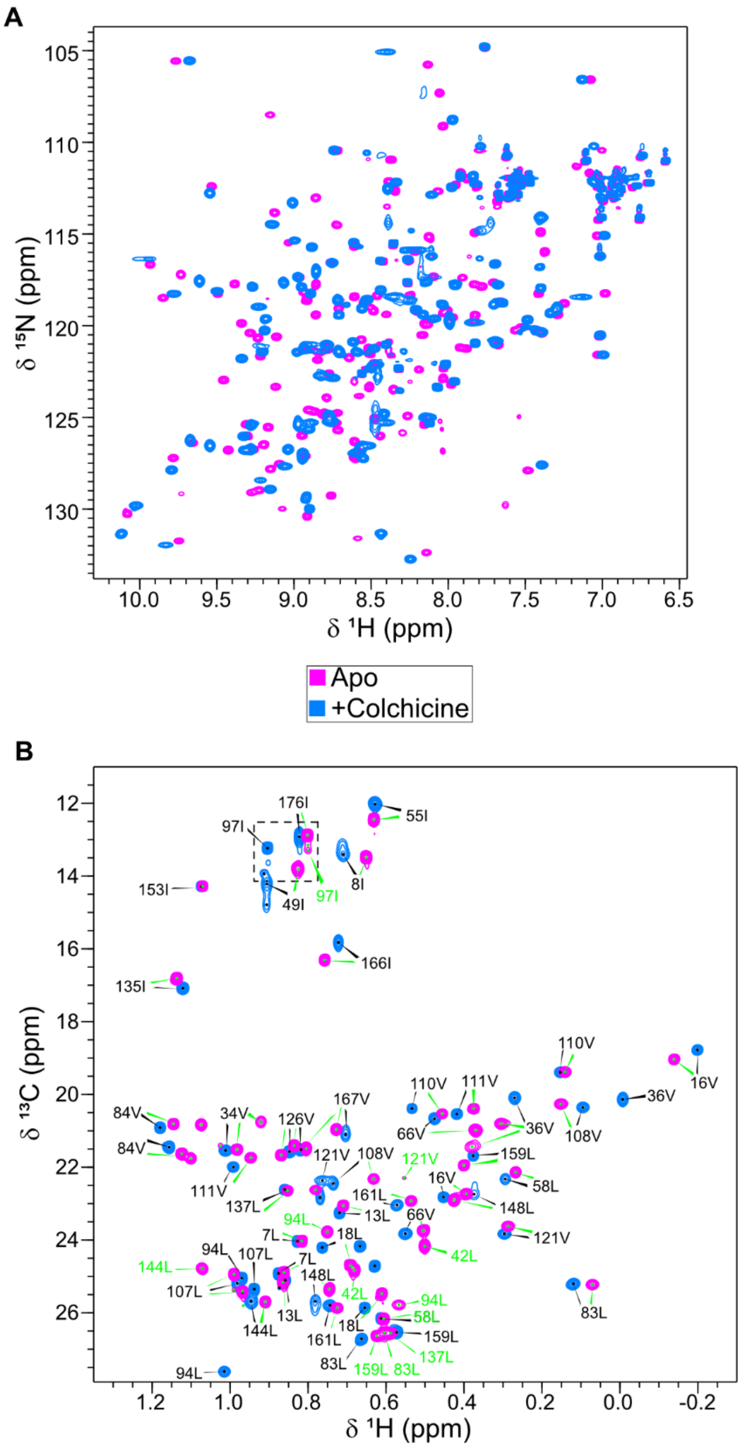

Figure S2. (A) Overlay of $^{1}H,^{15}N$ HSQC NMR spectra for the apo state Colchicalin (magenta) and the colchicine-bound complex (blue). (B) Overlay of $^{1}H,^{13}C$ HMQC spectra for the apo state (magenta) and colchicine-bound Colchicalin (blue). Assignments are indicated with green arrows (apo state) and black arrows (colchicine-bound state). The dashed square indicates the zoom region shown in Figure 3A. Spectra were recorded at 298 K and 600 MHz (^{1}H frequency).

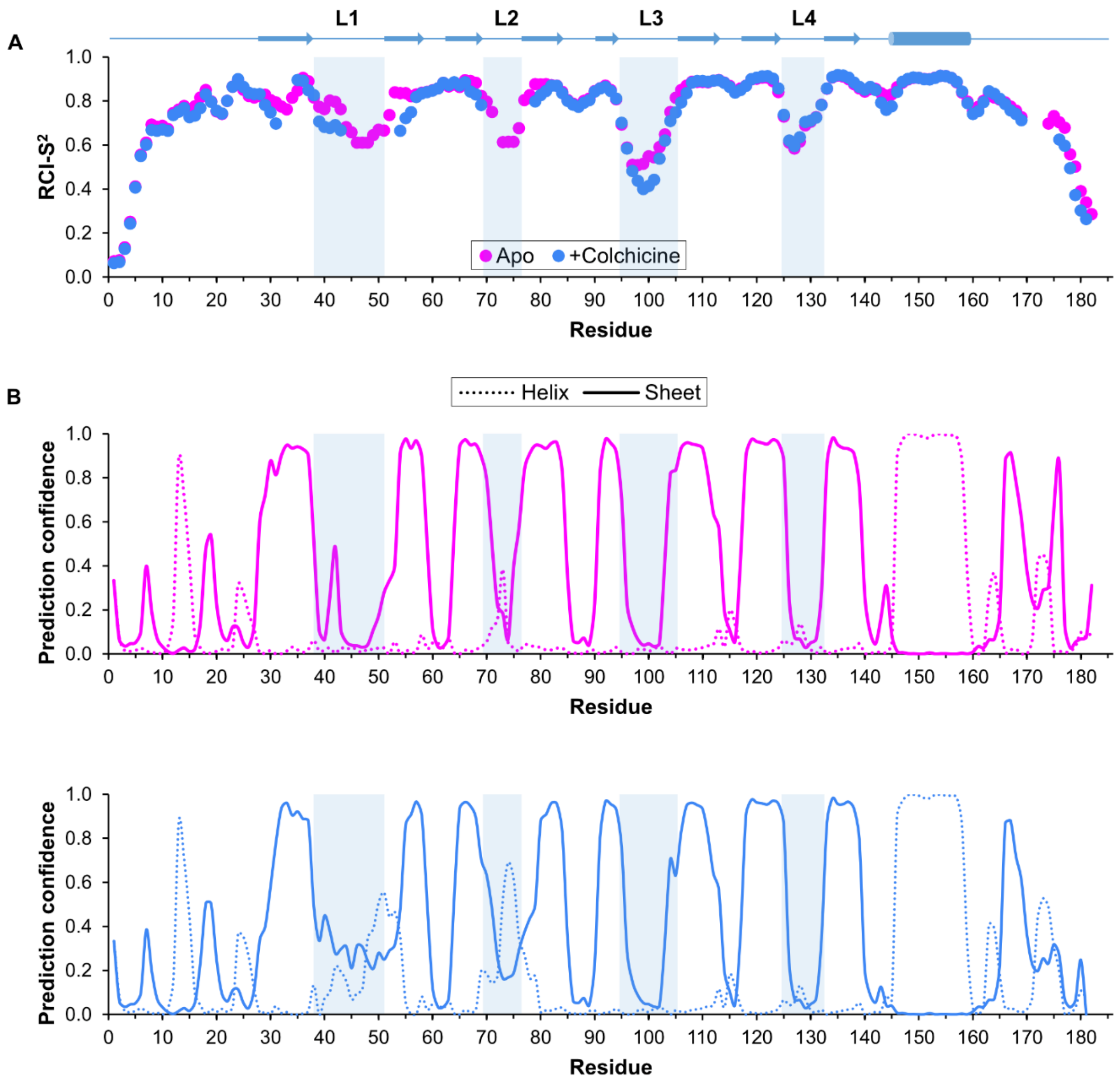

Figure S3. Secondary structure and S^2 order parameter prediction. Talos-N[32] was used with backbone chemical shifts (HN, N, C, CA, CB) to predict (A) the RCI-S^2 values[33] and (B) the secondary structure propensity for α-helix (dashed line) or β-sheet (solid line) for apo (pink) and colchicine-bound (blue) Colchicalin. Secondary structure elements are defined according to the structural comparison in Achatz *et al*.[31] and shown as a cartoon above the plots.

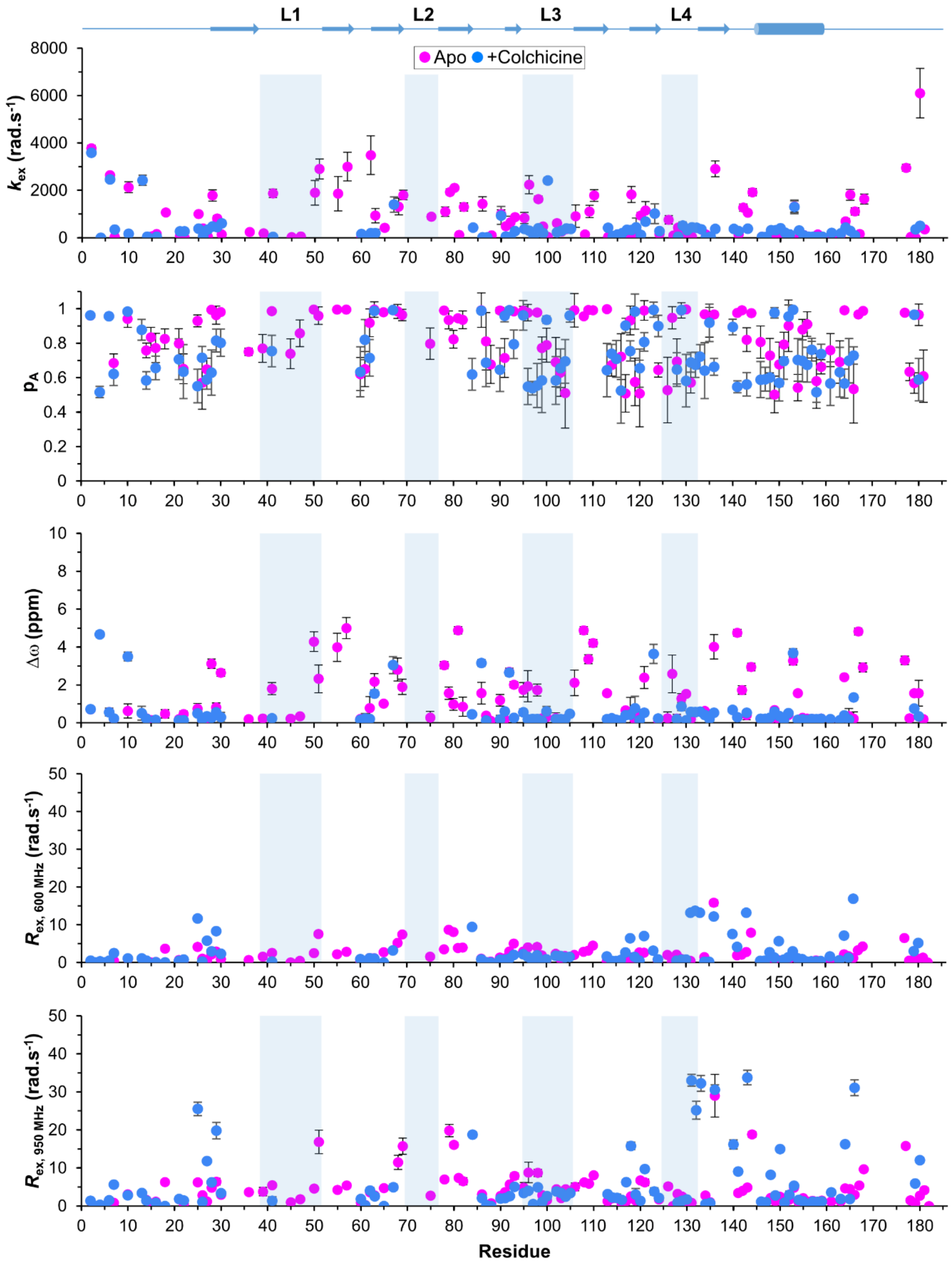

Figure S4. Parameters for microsecond-to-millisecond timescale backbone dynamics for apo and colchicine-bound Colchicalin. ^{15}N CPMG relaxation dispersion profiles recorded at 600 MHz and 950 MHz (^{1}H frequency) were fitted to slow-timescale motional models (see SI Methods). The fitted parameters k_{ex} (rad.s^{-1}), p_A (fraction) and Δω (ppm) as well as the extracted R_{ex} (rad.s^{-1}) rates ($R_{2,eff}$(1000 Hz) – $R_{2,eff}$(50 Hz)) are shown for apo (magenta) and colchicine-bound (blue) Colchicalin. Secondary structure elements are defined according to the structural comparison in Achatz *et al*.[31] and shown as a cartoon above the plots.

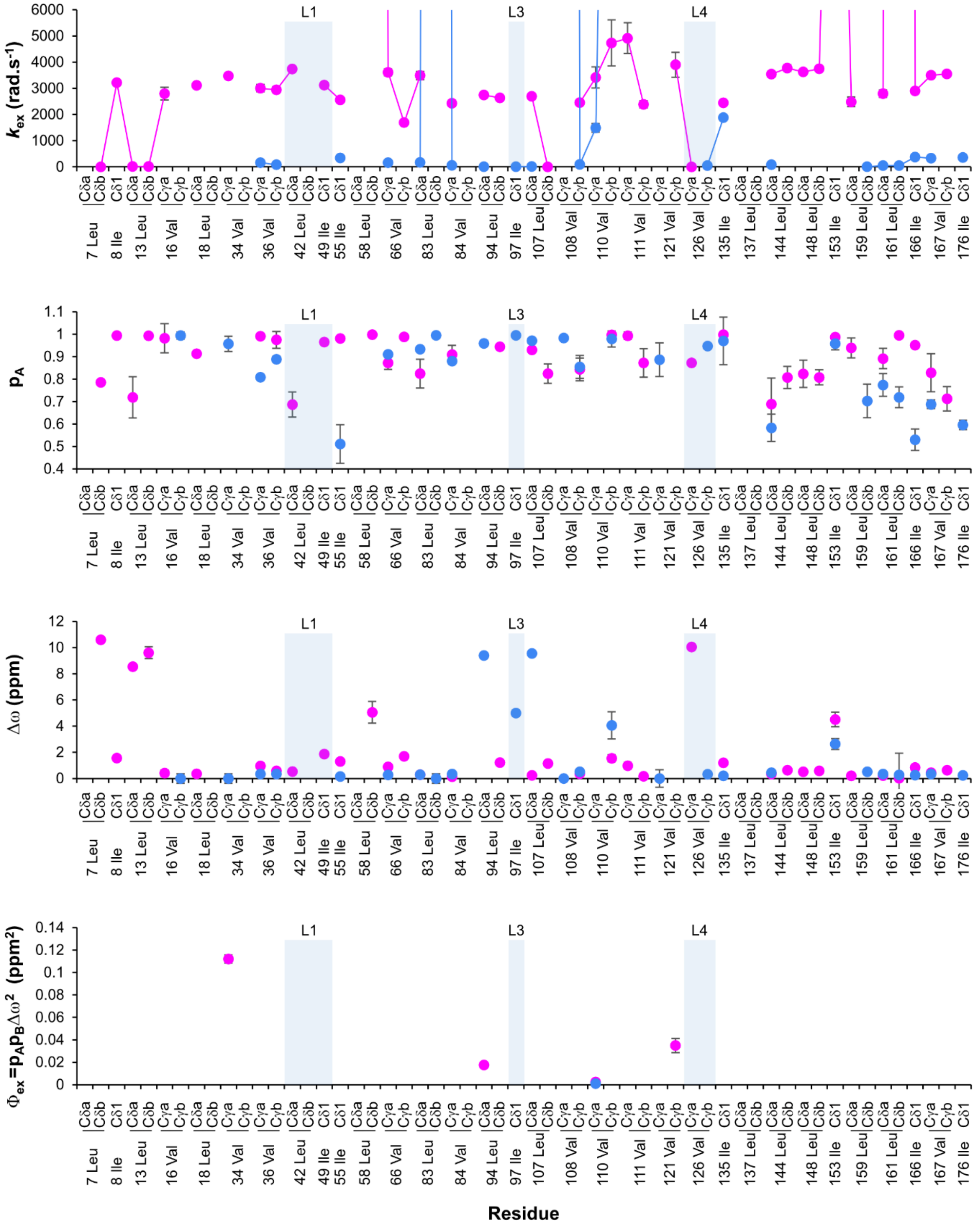

Figure S5. Parameters for sidechain microsecond-to-millisecond timescale backbone dynamics for apo and colchicine-bound Colchicalin. ^{13}C CPMG relaxation dispersion profiles recorded at 600 MHz and 950 MHz (^{1}H frequency) and 298 K were fitted to slow-timescale motional models (see SI Methods). The fitted parameters k_{ex} (rad.s^{-1}), p_A (fraction), Δω (ppm) and Φ_{ex} (ppm^2)[17] are shown for apo (magenta) and colchicine-bound (blue) Colchicalin.

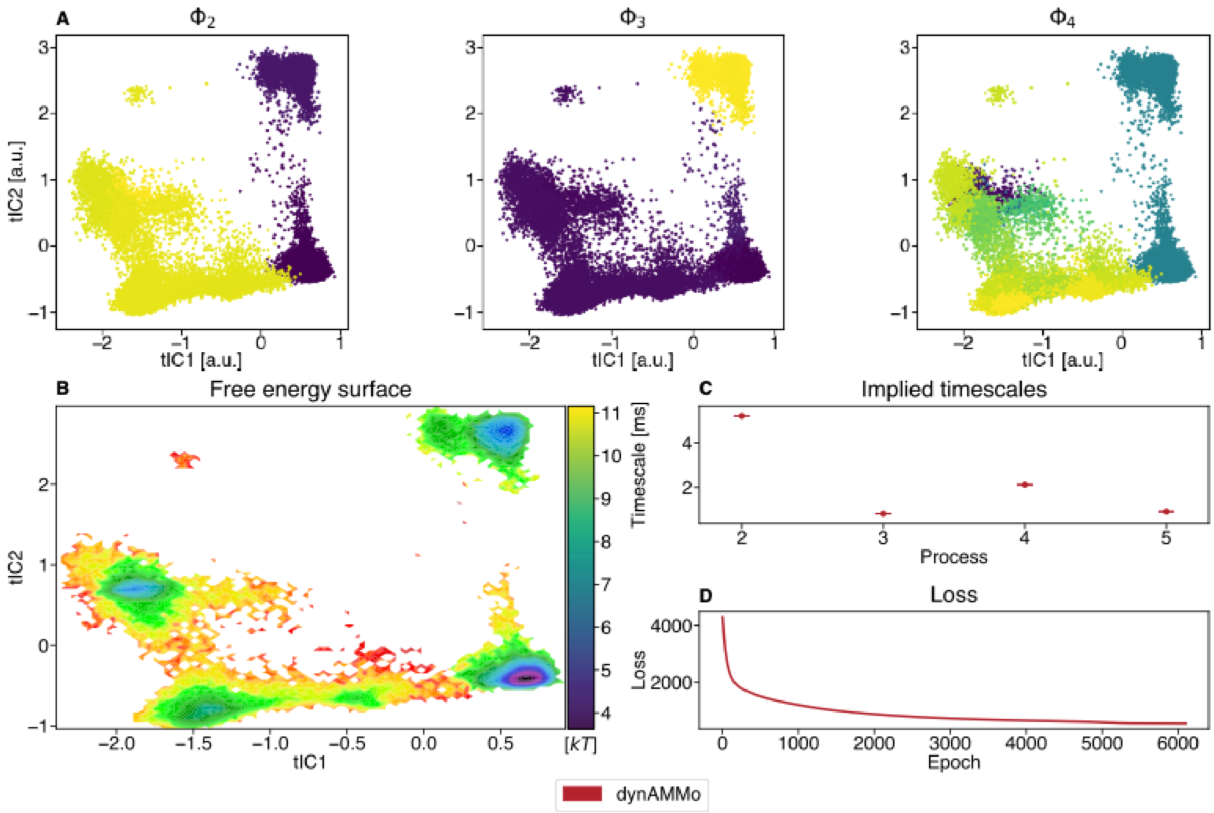

Figure S6. Overview of the dynamic Augmented Markov Model for Colchicalin. (A) The first three eigenfunctions are shown projected onto the two slowest time-lagged independent components (tICs). (B) The free energy surface of the model is shown. The colour coding corresponds to the colour bar shown on the right. (C) Timescales of the four slowest processes are shown in ms. (D) Loss function of the algorithm.

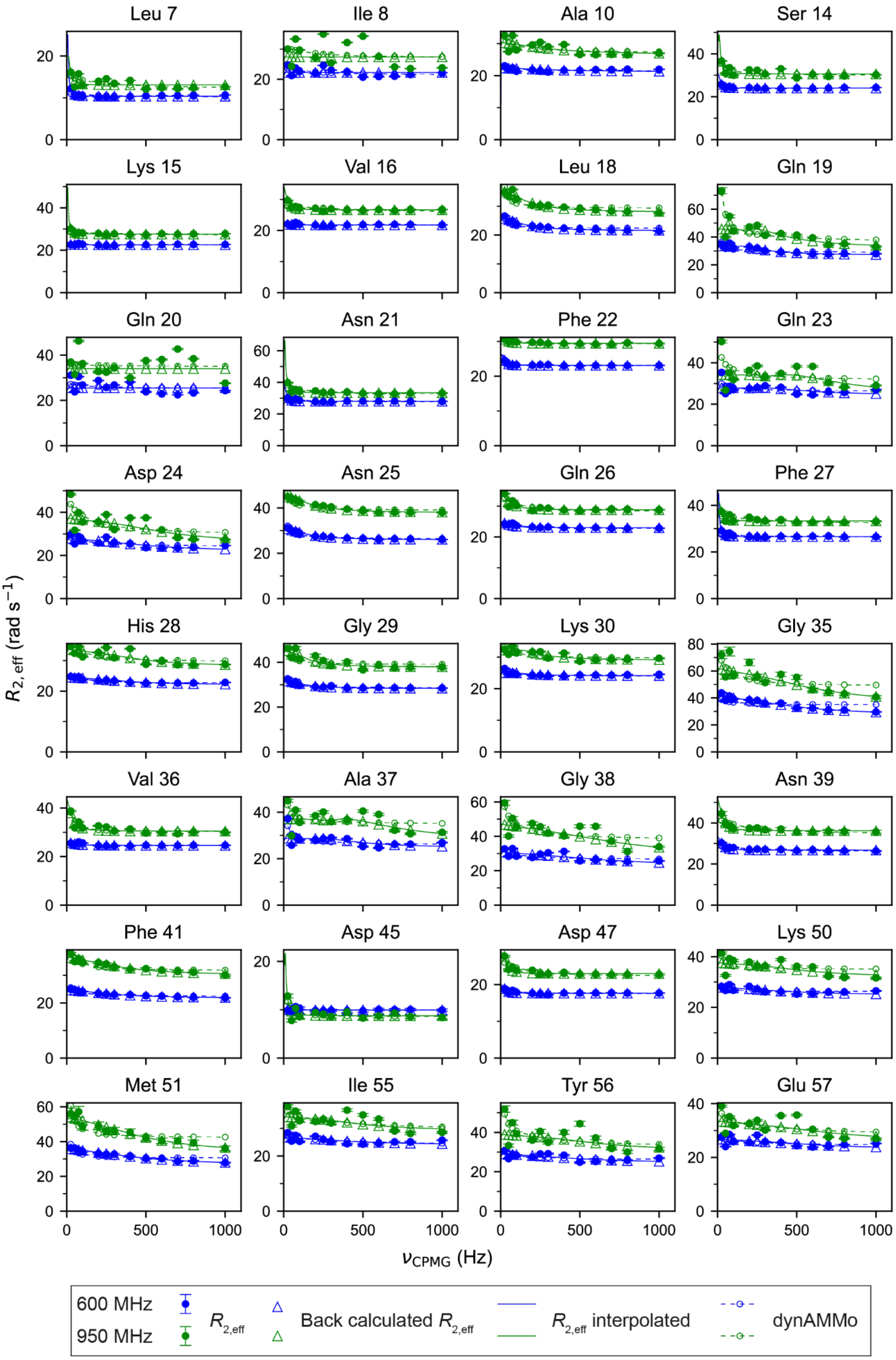
Leu 7
Ile 8
Ala 10
Ser 14
Lys 15
Val 16
Leu 18
Gln 19
Gln 20
Asn 21
Phe 22
Gln 23
Asp 24
Asn 25
Gln 26
Phe 27
His 28
Gly 29
Lys 30
Gly 35
Val 36
Ala 37
Gly 38
Asn 39
Phe 41
Asp 45
Asp 47
Lys 50
Met 51
Ile 55
Tyr 56
Glu 57
$R_{2,\mathrm{eff}}$ (rad s^{-1})
ν_{CPMG} (Hz)
600 MHz
950 MHz
$R_{2,\mathrm{eff}}$
Back calculated $R_{2,\mathrm{eff}}$
$R_{2,\mathrm{eff}}$ interpolated
dynAMMo

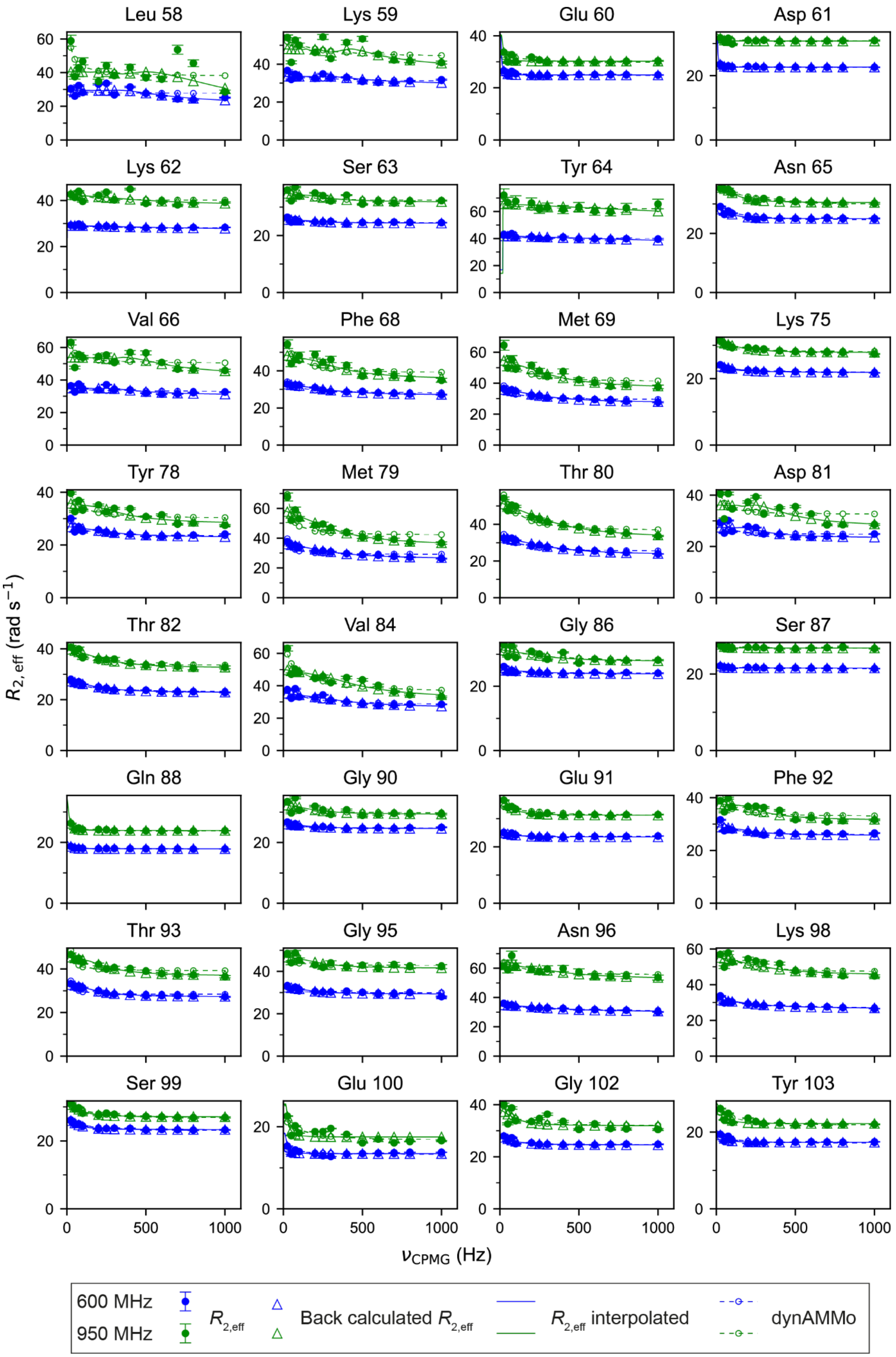

Leu 58
Lys 59
Glu 60
Asp 61
Lys 62
Ser 63
Tyr 64
Asn 65
Val 66
Phe 68
Met 69
Lys 75
Tyr 78
Met 79
Thr 80
Asp 81
Thr 82
Val 84
Gly 86
Ser 87
Gln 88
Gly 90
Glu 91
Phe 92
Thr 93
Gly 95
Asn 96
Lys 98
Ser 99
Glu 100
Gly 102
Tyr 103
$R_{2,\mathrm{eff}}$ (rad s^{-1})
ν_{CPMG} (Hz)
600 MHz
950 MHz
$R_{2,\mathrm{eff}}$
Back calculated $R_{2,\mathrm{eff}}$
$R_{2,\mathrm{eff}}$ interpolated
dynAMMo

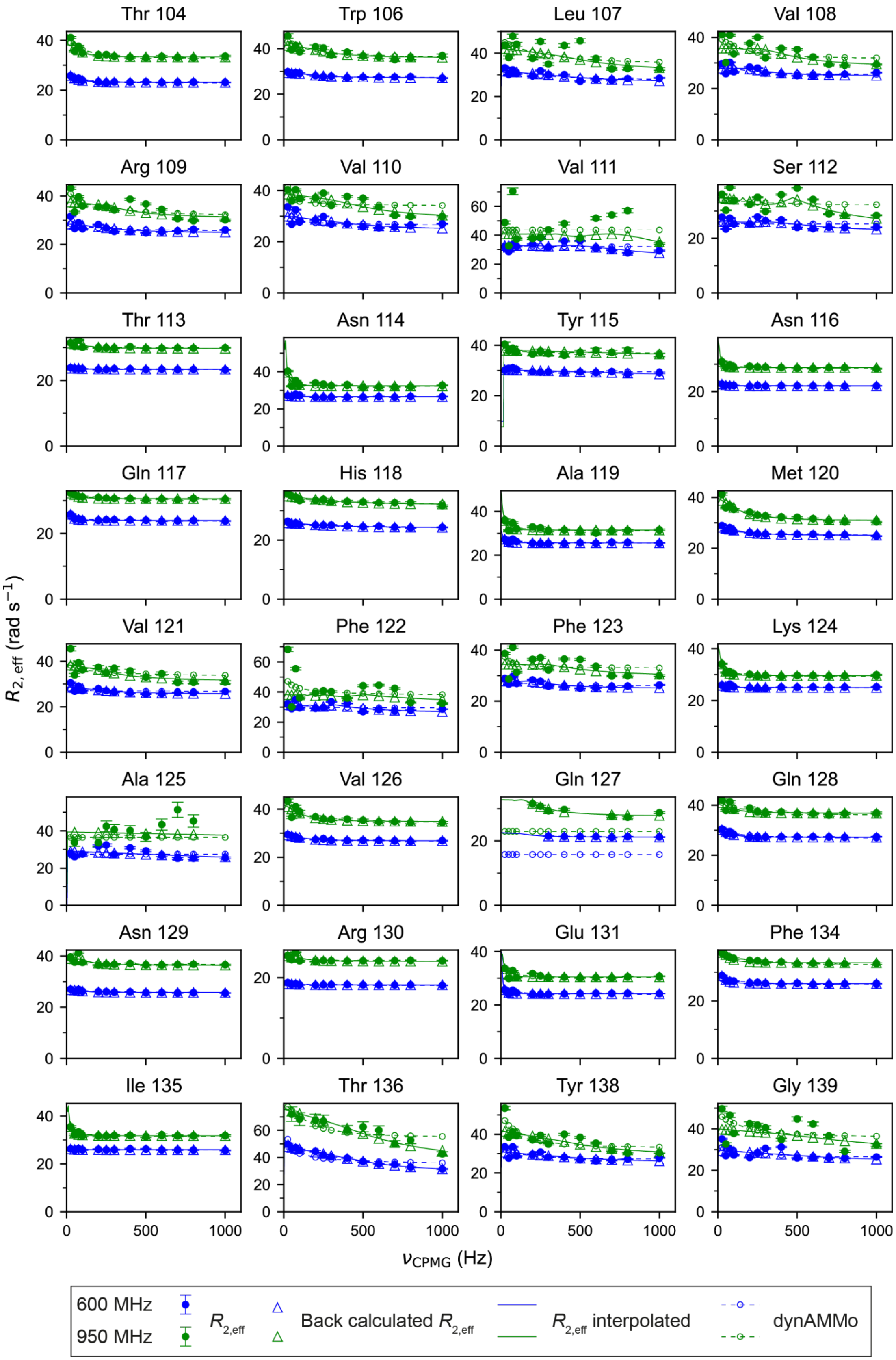
Thr 104
Trp 106
Leu 107
Val 108
Arg 109
Val 110
Val 111
Ser 112
Thr 113
Asn 114
Tyr 115
Asn 116
Gln 117
His 118
Ala 119
Met 120
Val 121
Phe 122
Phe 123
Lys 124
Ala 125
Val 126
Gln 127
Gln 128
Asn 129
Arg 130
Glu 131
Phe 134
Ile 135
Thr 136
Tyr 138
Gly 139
$R_{2,\mathrm{eff}}$ (rad s^{-1})
ν_{CPMG} (Hz)
600 MHz
950 MHz
$R_{2,\mathrm{eff}}$
Back calculated $R_{2,\mathrm{eff}}$
$R_{2,\mathrm{eff}}$ interpolated
dynAMMo

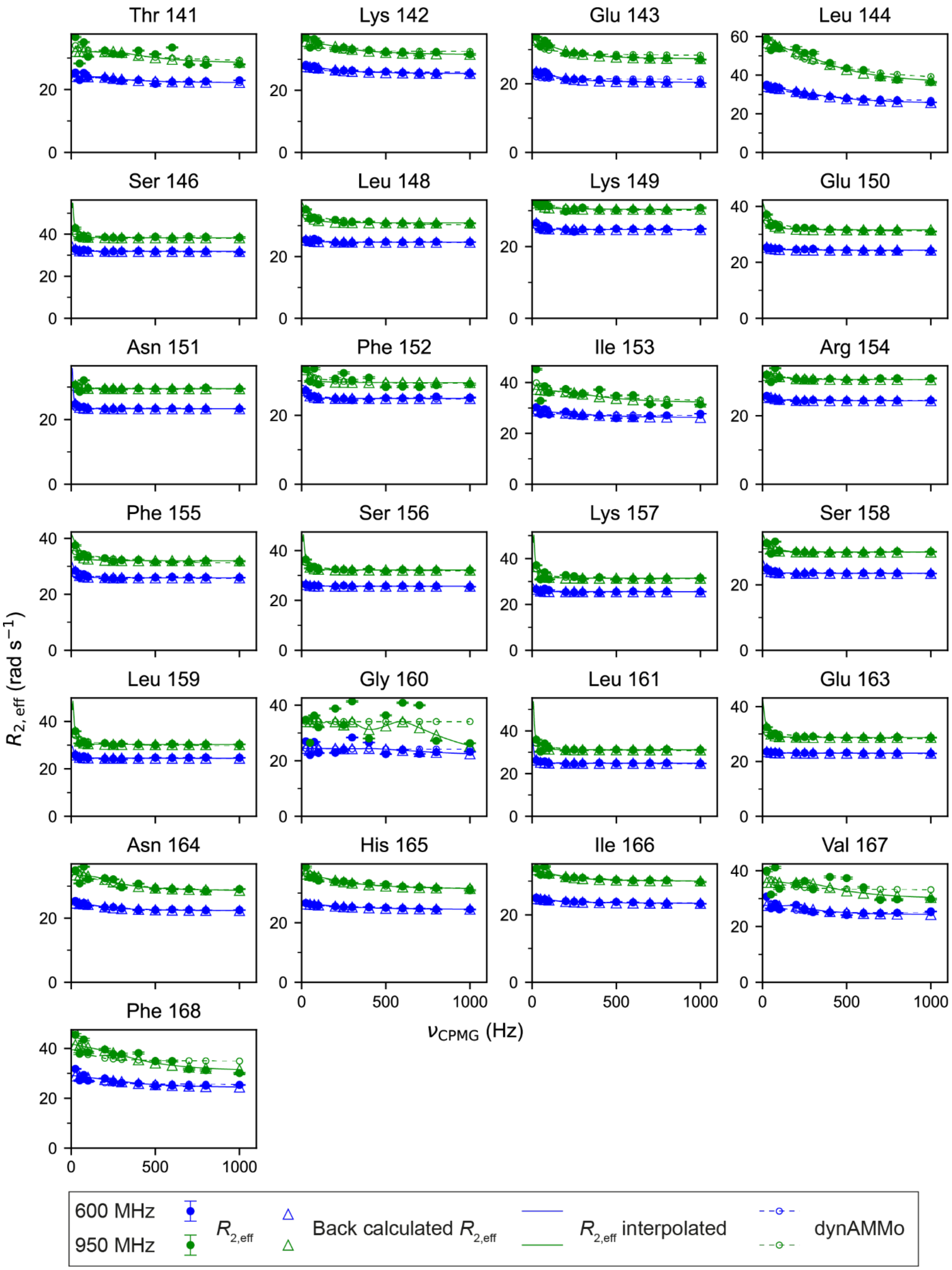

Figure S7. dynAMMo fits overlaid with experimental ^{15}N CPMG relaxation data for the apo state Colchicalin. $R_{2,\text{eff}}$ (rad s^{-1}) values are plotted as a function of ν_{CPMG} (Hz) for ^{15}N CPMG relaxation dispersion data recorded at 600 MHz (blue) and 950 MHz (green) (^{1}H frequency) respectively for the apo-state Colchicalin D6.2. Back calculated $R_{2,\text{eff}}$ values (triangles) and interpolated fits (solid lines) using models from the software Relax, described in the SI Methods, are shown. dynAMMo fits are shown as circles with dashed lines.

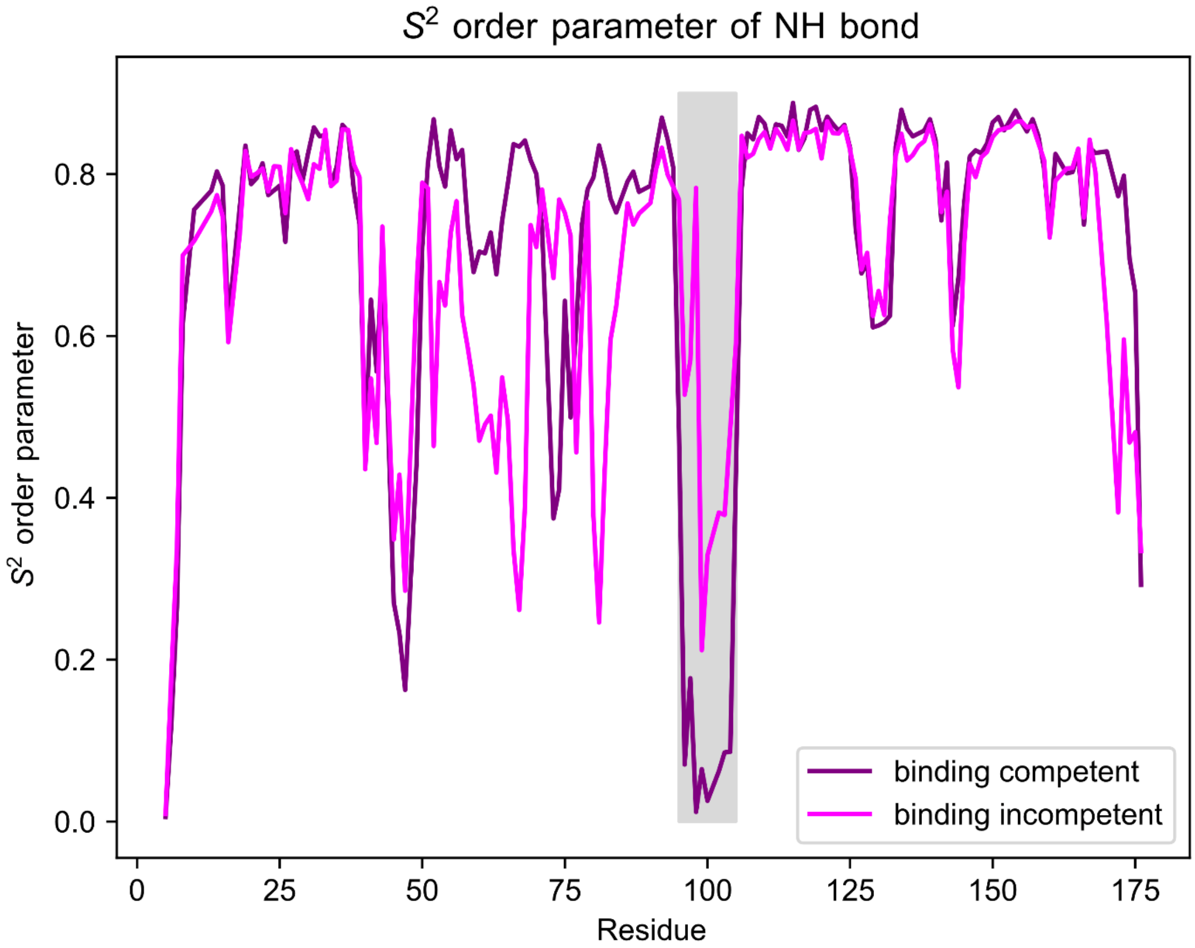

Figure S8. Comparison of fast internal motion using backbone S^2 order parameters for the apo-state Colchicalin. ^{15}N backbone order parameters for the binding-competent (pink) and binding-incompetent (purple) trajectories are shown as a function of residue numbers. The region corresponding to L3 is highlighted in a grey box.

Parameter	Value
lag	100 [ns]
α	-15 [a. u.]
β	1 [a. u.]
learning rate	1e-4 [a. u.]
learning rate (eigenfunctions)	1e-20 [a. u.]
learning rate (stationary distribution)	1e-20 [a. u.]

Table S1.
Overview of dynAMMo (hyper-)parameters

References

[1] M. Barkovskiy, E. Ilyukhina, M. Dauner, A. Eichinger, A. Skerra, *Biol. Chem.* **2019**, *400*, 351–366.
[2] A. Skerra, I. Pfitzinger, A. Plückthun, *Biotechnol. (N Y)* **1991**, *9*, 273–278.
[3] A. Skerra, *Gene* **1994**, *151*, 131–135.
[4] M. Sattler, J. Schleucher, C. Griesinger, *Prog. Nucl. Magn. Reson. Spectrosc.* **1999**, *34*, 93–158.
[5] M. J. Bostock, D. J. Holland, D. Nietlispach, *J. Biomol. NMR* **2012**, *54*, 15–32.
[6] W. F. Vranken, W. Boucher, T. J. Stevens, R. H. Fogh, A. Pajon, M. Llinas, E. L. Ulrich, J. L. Markley, J. Ionides, E. D. Laue, *Proteins* **2005**, *59*, 687–696.
[7] N. A. Farrow, R. Muhandiram, A. U. Singer, S. M. Pascal, C. M. Kay, G. Gish, S. E. Shoelson, T. Pawson, J. D. Forman-Kay, L. E. Kay, *Biochemistry* **1994**, *33*, 5984–6003.
[8] M. Gairí, A. Dyachenko, M. T. González, M. Feliz, M. Pons, E. Giralt, *J. Biomol. NMR* **2015**, *62*, 209–220.
[9] N.-A. Lakomek, J. Ying, A. Bax, *J. Biomol. NMR* **2012**, *53*, 209–221.
[10] M. Tollinger, N. R. Skrynnikov, F. A. A. Mulder, J. D. Forman-Kay, L. E. Kay, *J. Am. Chem. Soc.* **2001**, *123*, 11341–11352.
[11] C. Zwahlen, K. H. Gardner, S. P. Sarma, D. A. Horita, R. A. Byrd, L. E. Kay, *J. Am. Chem. Soc.* **1998**, *120*, 7617–7625.
[12] P. Lundström, P. Vallurupalli, T. L. Religa, F. W. Dahlquist, L. E. Kay, *J. Biomol. NMR* **2007**, *38*, 79–88.
[13] M. Bieri, E. J. d'Auvergne, P. R. Gooley, *J. Biomol. NMR* **2011**, *50*, 147–155.
[14] E. J. d'Auvergne, P. R. Gooley, *J. Biomol. NMR* **2008**, *40*, 107–119.
[15] E. J. d'Auvergne, P. R. Gooley, *J. Biomol. NMR* **2008**, *40*, 121–133.
[16] J. P. Carver, R. E. Richards, *J. Magn. Reson.* **1972**, *6*, 89–105.

[17] Z. Luz, S. Meiboom, *J. Chem. Phys.* **1963**, *39*, 366–370.
[18] R. Ishima, D. A. Torchia, *J. Biomol. NMR* **1999**, *14*, 369–372.
[19] E. J. d'Auvergne, P. R. Gooley, *J. Biomol. NMR* **2003**, *25*, 25–39.
[20] E. Jerschke, A. Eichinger, A. Skerra, *Acta Crystallogr. Sect. F Struct. Biol. Commun.* **2023**, *79*, 231–239.
[21] D. J. Price, C. L. Brooks, *J. Chem. Phys.* **2004**, *121*, 10096–10103.
[22] T. Darden, D. York, L. Pedersen, *J. Chem. Phys.* **1993**, *98*, 10089–10092.
[23] A. C. Simmonett, F. C. 4th Pickard, H. F. 3rd Schaefer, B. R. Brooks, *J. Chem. Phys.* **2014**, *140*, 184101.
[24] P. Eastman, J. Swails, J. D. Chodera, R. T. McGibbon, Y. Zhao, K. A. Beauchamp, L. P. Wang, A. C. Simmonett, M. P. Harrigan, C. D. Stern, R. P. Wiewiora, B. R. Brooks, V. S. Pande, **2017**, *13*, e1005659.
[25] C. Schütte, A. Fischer, W. Huisinga, P. Deuflhard, *A Hybrid Monte Carlo Method for Essential Molecular Dynamics*, **1998**.
[26] J. H. Prinz, H. Wu, M. Sarich, B. Keller, M. Senne, M. Held, J. D. Chodera, C. Schütte, F. Noé, *J. Chem. Phys.* **2011**, *134*, 174105.
[27] G. R. Bowman, V. S. Pande, F. Noé, Eds. , *An Introduction to Markov State Models and Their Application to Long Timescale Molecular Simulation*, Springer Netherlands, Dordrecht, **2014**.
[28] G. Pérez-Hernández, F. Paul, T. Giorgino, G. De Fabritiis, F. Noé, *J. Chem. Phys.* **2013**, *139*, 015102.
[29] M. Hoffmann, M. Scherer, T. Hempel, A. Mardt, B. de Silva, B. E. Husic, S. Klus, H. Wu, N. Kutz, S. L. Brunton, F. Noé, *Mach. Learn. Sci. Technol.* **2021**, *3*, 015009.
[30] C. Kolloff, S. Olsson, *Mach. Learn. Sci. Technol.* **2023**, *4*, 045050.
[31] S. Achatz, A. Jarasch, A. Skerra, *J. Struct. Biol. X* **2022**, *6*, 100054.
[32] Y. Shen, A. Bax, *J. Biomol. NMR* **2013**, *56*, 227–241.
[33] M. V. Berjanskii, D. S. Wishart, *J. Am. Chem. Soc.* **2005**, *127*, 14970–14971.
